# Consequences of hidden kinetic pathways on supramolecular polymerization†
†Electronic supplementary information (ESI) available. See DOI: 10.1039/d0sc02115f


**DOI:** 10.1039/d0sc02115f

**Published:** 2020-06-02

**Authors:** Jonas Matern, Kalathil K. Kartha, Luis Sánchez, Gustavo Fernández

**Affiliations:** a Organisch-Chemisches Institut , Westfälische Wilhelms-Universität Münster , Corrensstraße 36 , 48149 Münster , Germany . Email: fernandg@uni-muenster.de; b Departamento de Química Orgánica , Facultad de Ciencias Químicas, Universidad Complutense de Madrid , Ciudad Universitaria s/n , 28040 Madrid , Spain

## Abstract

In recent years, the development of sophisticated analytical tools, kinetic models and sample preparation methods has significantly advanced the field of supramolecular polymerization, where the competition of kinetic *vs.* thermodynamic processes has become commonplace for a wide range of building blocks. Typically, the kinetic pathways are identified in thermally controlled assembly experiments before they ultimately evolve to the thermodynamic minimum. However, there might be cases where the identification and thus the assessment of the influence of kinetic aggregates is not trivial, making the analysis of the self-assembly processes a hard task. Herein, we demonstrate that “hidden” kinetic pathways can have drastic consequences on supramolecular polymerization processes, to the point that they can even overrule thermodynamic implications. To this end, we analyzed in detail the supramolecular polymerization of a chiral Pd^II^ complex **1** that forms two competing aggregates (**Agg I** and **Agg II**) of which kinetic **Agg II** is formed through a “hidden” pathway, *i.e.* this pathway is not accessible by common thermal polymerization protocols. The hidden pathway exhibits two consecutive steps: first, **Agg II** is formed in a cooperative process, which subsequently evolves to clustered superstructures driven by rapid kinetics. At standard conditions, **Agg II** displays an extraordinary kinetic stability (>6 months), which could be correlated to its cooperative mechanism suppressing nucleation of thermodynamic **Agg I**. Furthermore, the fast kinetics of cluster formation sequester monomers from the equilibria in solution and prevents the system from relaxing into the thermodynamic minimum, thus highlighting the key implications of hidden pathways in governing supramolecular polymerization processes.

## Abstract 2

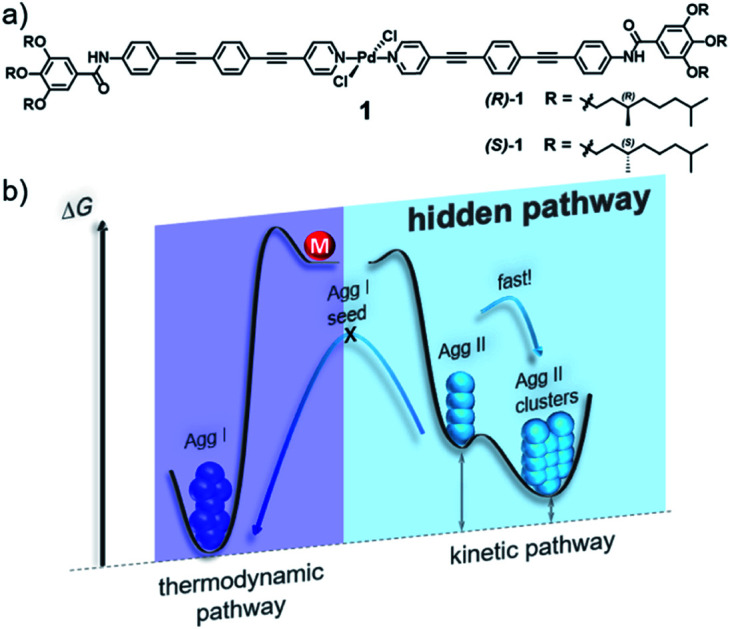
We show that hidden kinetic states have a dramatic impact on the thermodynamics of supramolecular polymerization, enabling new aggregation pathways with potentially new functionalities.

## Introduction

Complex self-assembly phenomena in nature often operate under far-from-equilibrium conditions and are governed by a fine balance of kinetic processes.^1^,^2^ Unravelling the subtle interplay of kinetic and thermodynamic pathways in self-assembled systems is therefore a prerequisite towards mimicking natural complexity.^3^–^5^ The past two decades have witnessed a significant advancement in the field of supramolecular polymerization, with focus being devoted to thermodynamically controlled systems.^6^,^7^ In recent years, however, the development of sophisticated analytical techniques, sample manipulation protocols and theoretical models has enabled the identification of competing kinetic pathways,^8^ which are inherent to virtually all types of building blocks. Therefore, understanding the interplay between such underlying kinetic *vs.* thermodynamic aggregation pathways is key to isolate supramolecularly pure species, which in turn determine the final functional properties of the materials.

For the vast majority of supramolecular polymers, competing kinetic pathways can be readily detected spectroscopically, but ultimately evolve to the thermodynamically stable species over an experimentally observable time scale.^3^,^9^–^17^ In some cases, this transformation can be accelerated by mixing the kinetic product with seeds of the thermodynamic assembly, thereby inducing controlled growth in a living manner.^3^,^10^,^12^,^13^,^18^ Only in rare cases, no spontaneous relaxation into the thermodynamic minimum occurs due to the close energy of kinetic and thermodynamic species^19^ or the lack of active polymerization termini.^20^

Generally, supramolecular polymerization processes are initiated when a hot monomer solution of a given building block in a suitable solvent is cooled down under controlled conditions. During cooling, kinetically controlled assemblies are the first to form and, subsequently, transform to the thermodynamic product either upon further cooling^21^–^25^ or over time at a constant temperature.^3^,^9^–^15^,^17^ Thus, the onset of the kinetic polymerization always precedes the thermodynamic process. This is logical considering that kinetic assemblies are typically formed through an isodesmic mechanism, which does not require to overcome a critical temperature/concentration. There are also few exceptions of kinetic assemblies that are formed *via* the cooperative mechanism,^14^,^15^ but they can be likewise obtained as intermediate species in cooling experiments prior to the formation of the thermodynamic product due to their higher elongation temperature (*T*_e_). Therefore, for most reported systems with coupled polymerization equilibria, kinetic states can be accessed by thermal polymerization protocols, specifically by variation of the cooling rate. Increasing the cooling rate, *i.e.* lowering the equilibration time, promotes the faster (→kinetic) processes and therefore, lowers *T*_e_ for the thermodynamic pathway (*T*therme). Once it falls below the temperature necessary to activate the kinetic pathway (*T*kine), an experimental identification of the corresponding kinetic species is possible.^12^,^13^,^19^,^26^ However, in contrast to all reported examples of cooperative kinetic pathways, a second case might also be possible if *T*therme >> *T*kine. Under these circumstances, it might not be possible to set the rate fast enough to access the kinetic pathway and the thermodynamic one will always dominate. In such cases, the kinetic process will be “hidden”, a term that has recently been used in the context of multicomponent seeded-growth.^27^ Therefore, the existence of hidden pathways may have a significant, but otherwise unrecognized influence on the overall characteristics of the systems, making necessary a detailed analysis of these phenomena.

**Fig. 1:**
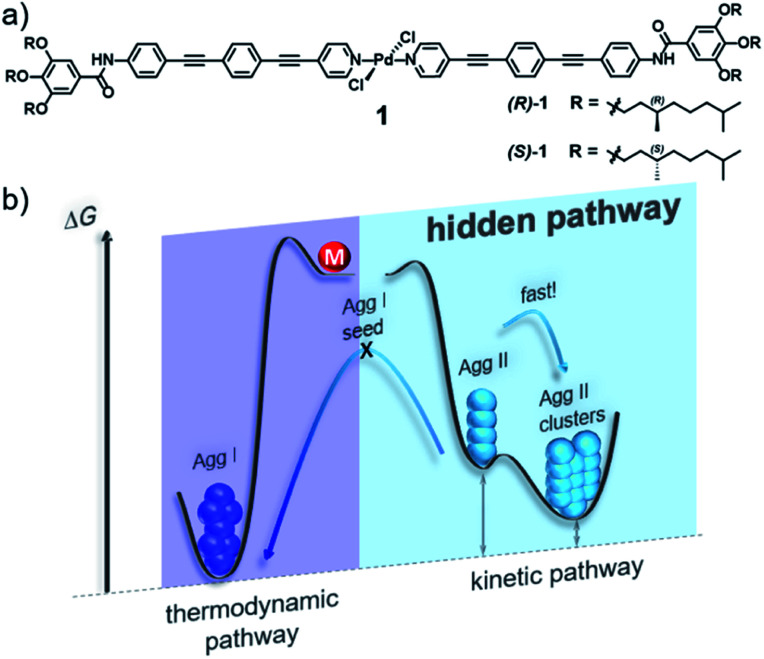
(a) Chemical structure of OPE-based bispyridyldichlorido Pd^II^ complex **1**. (b) Energy landscape outlining its complex self-assembly behaviour that incorporates a hidden pathway.

In this work, we demonstrate that hidden kinetic pathways can indeed have drastic consequences on the thermodynamics of supramolecular polymerization processes. In this context, we have investigated in detail the supramolecular polymerization of a chiral bispyridyldichlorido Pd^II^ complex **1** (Fig. 1a), the (*S*)-isomer of which was suggested in our preliminary work to form a single aggregate species.^28^ By doing so, we discovered a hidden, kinetic, cooperative state that is not accessible in its pure form by means of thermally controlled supramolecular polymerization, due to a lower *T*_e_ than the thermodynamic species. The hidden pathway incorporates two consecutive species, **Agg II** and its rapidly forming superstructures (**Agg IIc**, Fig. 1b), which have a high kinetic stability (>6 months). This stability and the fast kinetics of the hidden pathway prevent the system to relax into the thermodynamic minimum, thus highlighting the relevance of hidden pathways in governing supramolecular polymerization processes.

## Results and discussion

### Complex supramolecular polymerization behaviour of **1**

**Fig. 2:**
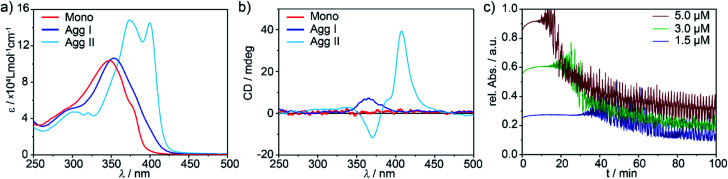
UV-Vis (a) and CD spectra (b) of (*R*)-**1** recorded upon cooling a monomer solution from 363 K (red spectra) to 293 K (blue spectra → **Agg I**) and upon solvophobic quenching (cyan spectra → **Agg II**) at *c* = 5 × 10^–6^ M. (c) Time-dependent evolution of **Agg II** (UV-Vis, *λ* = 399 nm) after injection of molecularly dissolved **1** in CHCl_3_ into MCH at different final concentrations. At higher concentrations, the onset of the clustering process is shifted to shorter lag times, indicating a consecutive pathway.

Self-assembled structures of d^8^ metal complexes exhibit particularly interesting photophysical properties^29^–^33^ and the presence of the metal fragment often enables multiple competing non-covalent interactions, which is beneficial to create kinetic aggregates.^34^–^37^ Preliminary self-assembly studies of the (*S*)-enantiomer of Pd complex **1**, however, suggested the presence of a single supramolecular species if monomer solutions in methylcyclohexane (MCH) were cooled from 363 K to 283 K at a rate of 1 K min^–1^.^28^ Herein, we re-investigated in detail the supramolecular polymerization of the (*R*)-enantiomer of **1**, using the previously reported (*S*)-**1** as model compound to generalize our findings. As both enantiomers exhibit an identical behaviour (except for the opposite sign of the CD signals, see Fig. S1c–f†), we will particularly focus our attention on (*R*)-**1**. Initially, temperature-dependent absorption studies were recorded, screening a broad range of cooling rates (10 to 0.1 K min^–1^; Fig. S2†). For all experiments, both enantiomers behave identically: the monomer absorption band at 348 nm (red plot in Fig. 2a, ^1^IL (π → π*) with possible ^1^MLCT contribution, for details see Fig. S3†) undergoes a slight bathochromic shift to 355 nm upon aggregation, which is concomitant with the expansion of the absorption band at higher wavelengths (blue plot in Fig. 2a, S1 and S2†). Thus, cooling of hot monomer solutions in MCH always leads to the same aggregate species (in the following denoted as **Agg I**), regardless of the cooling rate.

These results are somewhat surprising, considering that our molecular design incorporates various polarized heteroatoms that are theoretically able to engage in different types of competing non-covalent interactions. Therefore, we sought to investigate the possibility of “hidden” polymerization pathways by inducing more drastic changes in the experimental conditions. In fact, both thermal quenching (rapid cooling of hot monomeric MCH solutions to 273 K) and solvophobic quenching (injection of monomers dissolved in a good solvent, CHCl_3_, into an excess of aggregation-inducing solvent, MCH) led to remarkably different spectroscopic characteristics. UV-Vis spectroscopy revealed a second aggregate species (in the following termed as **Agg II**) with absorption maxima centred at 375 nm and 399 nm (Fig. 2 and S1†). These transitions are not only shifted to higher wavelengths than both monomeric **1** and **Agg I**, but also the overall absorption intensity is higher (hyperchromism). Additionally, a characteristic fine structure with two low-intensity maxima around *λ* = 320 nm and 300 nm is appreciable. This spectral pattern, particularly the low-energy absorption band at 399 nm, is characteristic for the formation of slipped π-stacks (*J*-aggregates), as previously observed for structurally related, OPE-based, discrete d^8^ metal complexes.^19^,^38^,^39^

A particularly interesting observation is the fact that only solvophobic quenching is able to create **Agg II** in its pure form, whereas thermal quenching always produces a minor amount of **Agg I** that coexists with **Agg II** (see UV-Vis and AFM studies in Fig. S1a and S4†). These findings suggest the presence of two supramolecular polymers that are close in energy, *i.e.* supramolecular polymorphs.^19^

The supramolecular polymerization of (*R*)-**1** was further examined by CD studies (Fig. 2b, for comparison with (*S*)-**1**, see Fig. S1c–f†). **Agg I** is characterized by a weak positive CD absorption band centred at *ca.* 360 nm, which remains invariant over time. The CD spectrum of **Agg II**, in contrast, displays a sharp bisignate band with a maximum in dichroic response at 407 nm and a minimum at 370 nm. Over time, this CD signal is increasingly contaminated with linear dichroism (LD, Fig. S5†), indicating the occurrence of a further process (*e.g*. macroscopic alignment).^40^,^41^ This assumption was corroborated by monitoring the time-dependent UV-Vis absorption changes of **Agg II** at the characteristic wavelength of *λ*_max_ = 399, where a second, defined process represented by an exponential decay in absorbance sets in after a short lag time (Fig. 2c and S6†). This decrease is accompanied by oscillations in absorbance intensity, which can be attributed to significant scattering originating from the formation of macroscopic nanostructures (*i.e*. clusters). The observation of a lag time is related to the existence of an energy barrier between **Agg II** and the macroscopic, clustered structures (in the following denoted as **Agg IIc**). This defines the two species as separate energy minima in the energy landscape, representing non-transient states.

To shed light onto the consecutive or competitive relationship of the two species, kinetic measurements monitoring the **Agg II** → **Agg IIc** transformation were performed at different concentrations. As depicted in Fig. 2c, the onset of the clustering process is delayed upon decreasing concentration, indicating that the two states are linked *via* a consecutive, two-stage pathway.^8^ Furthermore, the UV-Vis spectra of **Agg IIc** and **Agg II** are similar (Fig. S7†) and the clustering can be reversed at low concentrations and upon mechanical agitation (shaking, sonication), resulting in the spectra of **Agg II** (Fig. S8†). Therefore, the transition from **Agg II** in solution to higher ordered superstructures is of hierarchical nature, as also observed for other π-systems with interesting properties.^17^,^20^,^23^

**Fig. 3:**
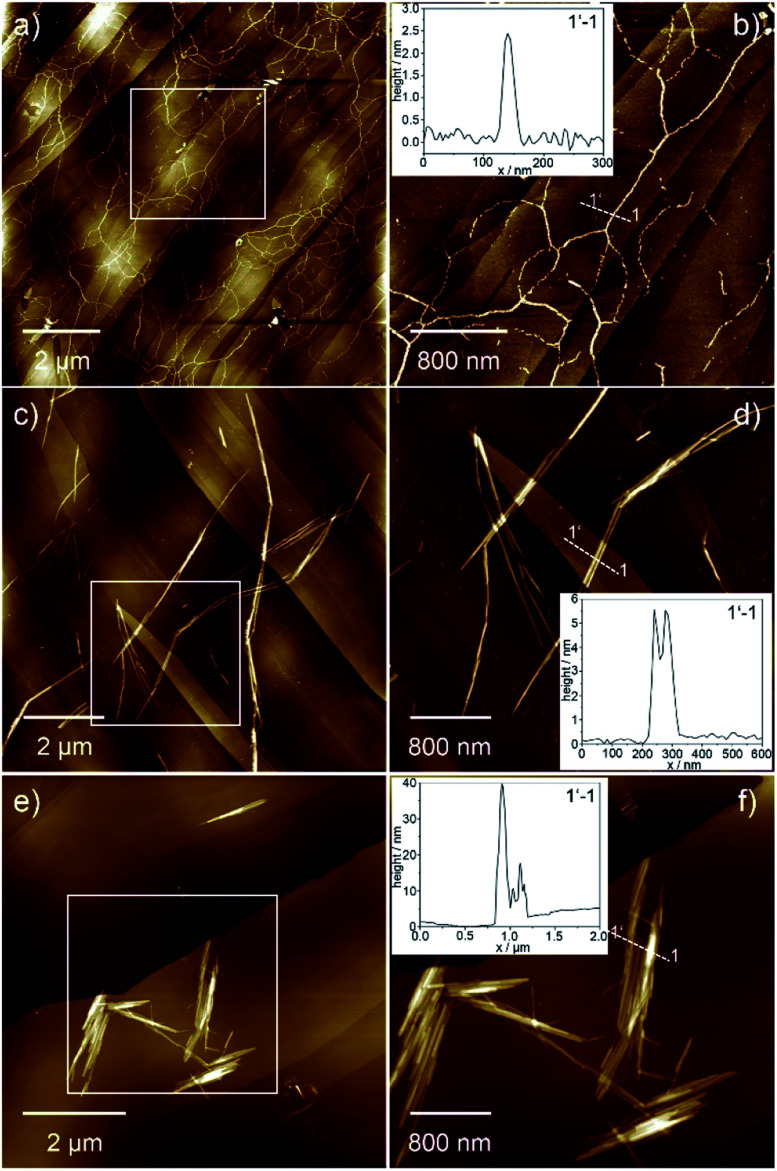
AFM images of **Agg I** (a and b), **Agg II** (c and d) and **Agg IIc** superstructures (e and f) obtained upon spin-coating the corresponding solutions onto HOPG. The insets show the height profile along the 1–1′ axis depicted in the images.

The morphology of the different aggregates could be visualized by atomic force microscopy (AFM, Fig. 3 and S4†) using highly ordered pyrolytic graphite (HOPG) as substrate. AFM analysis of **Agg I** displays a network of flexible fibres with a height of approx. 2.5 nm, a width of approx. 50 nm and several micrometres in length (Fig. 3a, b and S4e, f†). Single fibres merge and branch in some areas although most fibres exist as single strands that do not bundle. On the other hand, freshly prepared **Agg II** forms more rigid and polydisperse one-dimensional (1D) structures, which already exhibit some degree of lateral clustering as well as stacking along the *z* axis (Fig. 3c, d and S4g†). In contrast to **Agg I**, isolated aggregates are scarce for **Agg II**. Instead, greater structures consisting of several subunits are visualized. Several of such clusters exist spatially isolated from one another, however at this stage, no higher-ordered structures are developed.

AFM images of **Agg II** polymers obtained upon quenching and subsequent ageing (**Agg IIc**) could substantiate the formation of hierarchical superstructures from **Agg II** by strong clustering (Fig. 3e, f and S4h†). Very rigid agglomerates with a height of 10–50 nm, diameters up to 1 μm and lengths of several micrometres are formed. The shorter length of the aged assemblies (**Agg IIc**) compared to the aggregates obtained from freshly prepared solutions (**Agg II**) can be explained by the use of slight sonication prior to AFM analysis in order to prevent deposition of large superstructures onto the HOPG substrate (Fig. 3).

### Structural elucidation of the assemblies

**Fig. 4:**
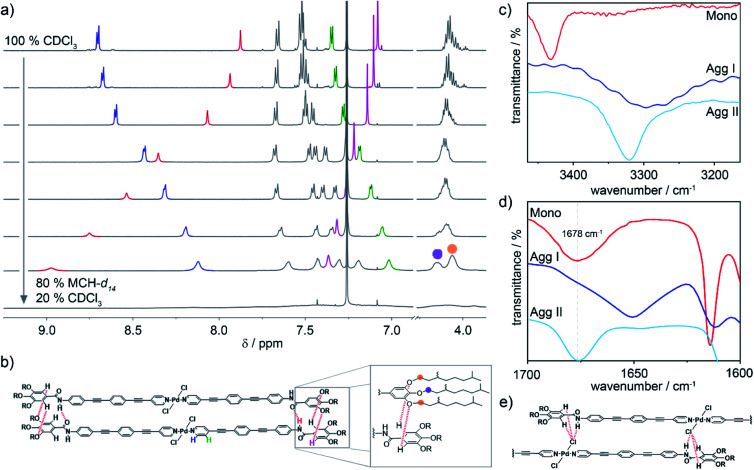
(a) Development of the ^1^H NMR resonances of (*R*)-**1** upon self-assembly into **Agg I**, induced by decreasing solvent polarity from 100% CDCl_3_ (monomer) to 20% CDCl_3_/80% MCH-*d*_14_ (*c* = 2 × 10^–3^ M). (b) Parallel molecular packing governed by amide-to-amide hydrogen bonding and unconventional C–H···O_alkoxy_ interactions, which are illustrated in the zoom. Right panel: partial FT-IR spectra of monomer (CHCl_3_, *c* = 0.5 mM), **Agg I** and **Agg II** (both thin films), showing regions of N–H (c) and carbonyl C=O (d) stretching frequencies. (e) Excerpt of the molecular packing in **Agg II** illustrating the N–H···Cl interactions leading to a slipped molecular arrangement.

In order to gain insights into the interaction patterns dictating the arrangement of the molecules within the different aggregates, ^1^H NMR measurements and Fourier-Transform Infrared (FT-IR) spectroscopy were carried out. Initially, the molecular packing of **Agg I** was probed by solvent-dependent ^1^H NMR spectroscopy (*c* = 2 × 10^–3^ M, *T* = 298 K), adding increasing volume fractions of MCH-*d*_14_ to CDCl_3_. Prior to these experiments, we demonstrated by means of UV-Vis spectroscopy under identical conditions, that this preparation method efficiently isolates **Agg I** (Fig. S9†). Fig. 4a depicts the evolution of the ^1^H NMR signals of **1** when increasing volume fractions of MCH-*d*_14_ (favouring the formation of **Agg I**) are added to molecularly dissolved **1** in CDCl_3_. As expected, the monomer-to-aggregate transition is accompanied by a loss of fine structure and broadening of all resonances. In the aromatic region, the most pronounced changes are observed for the amide N–*H* proton (red), the α- and β-proton of the pyridine moiety (blue and green, respectively) as well as the gallic C–*H* proton (pink). Aromatic interactions originating from the spatial proximity of the OPE units upon aggregation result in a significant upfield shift of the α- and β-pyridine protons of Δ*δ* = –0.58 ppm and –0.34 ppm, respectively. Additionally, the amide signal is strongly deshielded from 7.9 ppm to above 8.9 ppm (Fig. 4a), suggesting the formation of hydrogen bonds.^19^,^42^–^44^ Most strikingly, the resonance corresponding to the gallic protons is deshielded in contrast to all other aromatic signals. This is suggestive of the gallic proton interacting with an electron withdrawing moiety, most likely *via* an unconventional hydrogen bonding interaction (C_arom_–H···X).^38^,^43^,^45^ Furthermore, the single resonance corresponding to the peripheral O–C*H*_2_ methylene protons splits into two new resonances with a ratio of 2 : 1 (Fig. 4a). This indicates a different electronic environment for the methylene groups of the peripheral chains (orange circle in Fig. 4a and b) compared to those of the central chain (purple circle in Fig. 4a and b).

Based on the spectroscopic features, a parallel packing is proposed for **Agg I** (Fig. 4b). In this arrangement, adjacent alkoxybenzene moieties are spatially close and the aforementioned intermolecular C_arom_–H···X interactions can be formed between gallic protons and alkoxy oxygens. The proposed parallel arrangement is also supported by 2D ROESY NMR spectroscopy, where no interactions between the peripheral alkoxy chains and the central aromatic units could be detected (Fig. S10†).

To further elucidate the interaction patterns governing the formation of both aggregates, we subsequently conducted FT-IR spectroscopy studies of solutions and thin films of the respective aggregate and monomer (Fig. 4c and d). The equivalence of the supramolecular polymer structures in the drop-coated thin films to the polymers in solution was confirmed by UV-Vis spectroscopy (Fig. S7†). The FT-IR spectrum of **Agg I** reveals a defined N–H stretching band at 3296 cm^–1^ (Fig. 4c), indicating that the amide N–H groups are engaged in hydrogen bonding.^19^,^43^,^46^ Additionally, in the carbonyl region, the amide I stretching appears at 1650 cm^–1^ (Fig. 4d), a value that is diagnostic of hydrogen-bonded carbonyl groups.^43^,^46^,^47^ For **Agg II**, the N–H stretching frequency is characteristic for hydrogen-bonded groups as well (*ν*_N–H_ = 3315 cm^–1^), albeit with a weaker H-bonding strength compared to **Agg I**. However, in contrast to **Agg I**, the high wavenumber obtained for the amide I carbonyl stretching (*ν*_C=O_ = 1678 cm^–1^) suggests that the carbonyl groups are not participating in H-bonding. Theoretically, the only other groups capable to act as H-bond acceptors are either the Pd-bound chlorine substituents or the peripheral alkoxy oxygens. To disclose which of the two interactions (N–H···Cl *vs.* N–H···O_alkoxy_) is prevailing in **Agg II**, the C_aryl_–O band as well as the *meta*-substituted ring vibration were inspected. Notably, both aggregates are characterized by a single C_aryl_–O band (at 1215 cm^–1^ for **Agg I** and at 1208 cm^–1^ for **Agg II**; Fig. S11a†). The lack of band splitting, which is a distinctive feature of the interaction between the amide N–H proton of one molecule and one single alkoxy oxygen of a neighbouring one, points to an N–H···Cl interaction pattern for **Agg II**. Additionally, the band of the *meta*-substituted ring vibration is the same for both species (1063 cm^–1^, Fig. S11b†). This indicates that there is no major difference in the electronic environment of this atom in both packing modes, ruling out the possibility of an N–H···O_alkoxy_ interaction for **Agg II**. Accordingly, we propose that **Agg II** is stabilized by cooperative aromatic and N–H···Cl hydrogen bonding interactions, resulting in a slipped molecular arrangement with a lateral offset between molecules in the adjacent layers (Fig. 4e).

For **Agg I**, however, classical amide–amide hydrogen bonds alongside aromatic interactions facilitate a parallel arrangement of the chromophores. This molecular orientation explains the deshielding of the gallic protons *via* weak C_arom_–H···O_alkoxy_ contacts as well as the splitting of the alkoxy O–C*H*_2_ resonances in the NMR experiments (Fig. 4a and b). As only two out of three alkoxy oxygens per molecule side are involved in H-bonding, a 2 : 1 intensity ratio results. Such involvement of the aromatic protons of trialkoxybenzene moieties in hydrogen bonding has been previously observed for different supramolecular polymers.^19^,^38^,^43^,^45^,^48^

The distinct packing experimentally observed for **Agg I** and **Agg II** can furthermore explain not only the different tendency to form superstructures but also the dissimilar dichroic features. In **Agg I**, the parallel molecular orientation facilitates van der Waals interactions between adjacent alkoxy chains within the same polymer strand. This orientation results in a small rotation angle between the molecules in the aggregate which yields a weak CD response, even in the fully aggregated state (*α* ∼ 1). In contrast, the non-covalent forces operating in **Agg II** favour larger rotation angles in the stacked units to afford more intense dichroic signals. At the same time, an interstrand entanglement of the paraffinic side chains is more likely to occur for **Agg II**, ultimately leading to clustered superstructures.

### Mechanistic analysis: supramolecular polymorphism

In order to elucidate the relative stability of the three supramolecular polymers at ambient conditions, their evolution was monitored by time-dependent UV-Vis studies (Fig. S12†). To our surprise, apart from the expected transformation of **Agg II** into **Agg IIc** over time (Fig. 2c), neither **Agg I** nor **Agg IIc** undergo any significant spectral changes in the time course of six months. Only for **Agg II**, the transition at 399 nm becomes slightly more prominent at the expense of the maximum at 375 nm (Fig. S12†). This could originate from an increased macroscopic growth of the aggregates over time, under preservation of the packing mode.

**Fig. 5:**
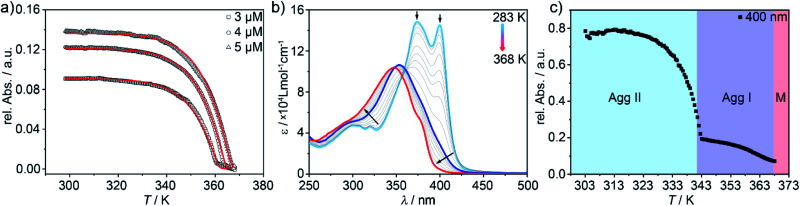
(a) Aggregation curves of **Agg I** (*λ* = 400 nm) upon cooling hot monomer solutions from 368 K to 298 K and fits to the nucleation-elongation model. (b) UV-Vis spectra recorded upon heating **Agg II** under constant mechanical agitation (*c* = 5 × 10^–6^ M). (c) Corresponding two-stage disassembly curve recorded at *λ* = 400 nm.

We next analysed the mechanism governing the formation of **Agg I** and **Agg II** by temperature-dependent UV-Vis studies. For **Agg I**, thermodynamic analysis of the experimental data obtained at the slowest cooling rate (0.1 K min^–1^; thermodynamic control) revealed a cooperative supramolecular polymerization (Fig. 5a). Fitting of the cooling curves to the nucleation-elongation model^49^ gave an average Gibbs free energy value of Δ*G* = –50.1 kJ mol^–1^ (for further data, see Table S1†). On the other hand, given that **Agg II** can only be isolated by thermal or solvophobic quenching, heating experiments of freshly prepared solutions of **Agg II** had to be applied to inspect the mechanism (Fig. 5b). In order to minimize the interference of cluster formation, the temperature-dependent UV-Vis experiments were carried out under continuous mechanical agitation. Initial heating of **Agg II** to 313 K did not cause any spectral changes, indicating that the polymer remains stable in this temperature window (Fig. 5b and c). However, subsequent heating above 313 K leads to a gradual depletion of the transition at 399 nm and a shift of the absorption maximum to 354 nm. These spectral features are diagnostic of **Agg I** (blue spectrum). Thus, the disassembly of **Agg II** proceeds *via* the formation of **Agg I**, which remains stable up to *ca.* 340 K (Fig. 5c). Further increasing the temperature initiates the disassembly of **Agg I**, as evident from the characteristic monomer absorption band centred at 348 nm obtained at high temperatures (Fig. 5b). The corresponding melting curve obtained from the data at 400 nm exhibits two stages corresponding to the transformation of **Agg II** to **Agg I** and subsequent disassembly into monomers (Fig. 5c).

The low-temperature regime of the disassembly curve (288 K to 352 K) could be fitted to the nucleation-elongation model (Fig. S13†), revealing that the disassembly of **Agg II** also occurs in a cooperative fashion. Surprisingly, the corresponding thermodynamic parameters indicated a higher stability of **Agg II** (Δ*G* = –64.6 kJ mol^–1^, Table S2†) compared to **Agg I**. However, the appearance of **Agg I** at intermediate temperatures is in stark contrast to **Agg II** being the more stable supramolecular polymer, as suggested by the thermodynamic parameters.

In order to address this discrepancy, complementary solvent-dependent denaturation studies were performed for both aggregates, by adding increasing volume fractions of a good solvent (CHCl_3_) to the respective aggregate solutions in MCH at a constant concentration (Fig. S14 and S15†).^50^ In analogy to the previous heating experiments, **Agg I** appeared as an intermediate species prior to full disassembly of **Agg II** into the monomer, as supported by UV-Vis and CD spectroscopy (Fig. S16†). Additionally, by monitoring its evolution over time, it could be proven that **Agg I** is not a transient state in the disassembly of **Agg II**, but rather a stable intermediate. Even 12 h after the addition of ∼12% v/v chloroform to a solution of **Agg II**, the UV-Vis and CD spectra of the emerging **Agg I** remained unchanged (Fig. S17†). Again, the cooperative model was suitable to fit the experimental data for both species. However, in contrast to the results obtained by temperature-dependent studies, the thermodynamic parameters extracted from denaturation experiments point to a lower stability of **Agg II** (Δ*G* = –41.6 kJ mol^–1^) in comparison to **Agg I** (Δ*G* = –51.5 kJ mol^–1^) under identical conditions. This small energy difference (∼10 kJ mol^–1^) between the two polymers lies within the range of systems to be considered polymorphs.^51^–^53^

Notably, both supramolecular polymers **Agg I** and **Agg II** are formed by a cooperative mechanism. Such dual-cooperative systems are rarely described in literature,^15^,^54^ as in most systems the kinetic species is formed in an isodesmic process or no clear assignation of either of the two mechanisms is possible. The contrasting results of the thermodynamic analysis, the intermediate formation of **Agg I** in temperature- and solvent-dependent experiments and the unusual dual-cooperative character of the self-assembly prompted us to investigate the relationship of **Agg I** and **Agg II** more deeply.

### Impact of the hidden pathway: kinetics overrule thermodynamics

**Fig. 6:**
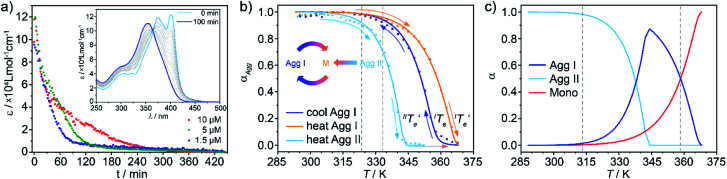
(a) Time-dependent evolution of the absorbance at 399 nm monitoring the **Agg II** → **Agg I** interconversion in thermal annealing experiments at 323 K, under constant mechanical agitation. The increase in transformation time upon increasing concentration reveals a competitive relationship between **Agg II** and **Agg I**. Inset: corresponding UV-Vis spectra recorded for *c* = 1 × 10^–5^ M, illustrating the transition to **Agg I**. (b) Plots of *α*_Agg_*vs. T* for cooling/heating processes of **Agg I** (blue/red) and heating process of **Agg II** (cyan) for a total concentration of *c* = 5 × 10^–6^ M (c) estimated molar fraction of free monomer, **Agg I** and **Agg II***versus* temperature.

The lack of evolution of **Agg I** and **Agg II**/**Agg IIc** at room temperature made it necessary to accelerate the systems kinetics in order to be able to monitor potential aggregate interconversion processes. This was achieved by annealing the solutions of both aggregates at elevated temperatures, which still lay below the respective elongation temperatures (*T*_e_) of both species. Time-dependent UV-Vis measurements at 323 K and 333 K showed no changes for **Agg I**, whereas **Agg II** converted to **Agg I** over time (Fig. 6a inset, Fig. S18 and S19†), as also corroborated by CD spectroscopy (Fig. S20†). These findings suggest a kinetically controlled supramolecular polymerization for **Agg II**, while **Agg I** represents the thermodynamic species of the solution equilibria. With increasing concentration, the **Agg II** → **AggI** transformation slows down (Fig. 6a), revealing the competitive nature of both aggregation pathways (**Agg I** and **Agg II**).

As the self-assembly of **1** incorporates both competitive and consecutive pathways, we assessed the influence of the consecutive, hierarchical clustering step on the kinetics and stability of the overall system. Considering the short lag time before significant light scattering is observed (Fig. 2c), it becomes obvious that the clustering process (*i.e.* topological change) proceeds with fast kinetics once polymers of type **Agg II** are formed in solution. Thus, the thermodynamic equilibration of the system is outrun by the fast kinetics of the clustering step in the hidden, kinetic pathway. Therefore, **Agg IIc** sequesters **Agg II** from the solution equilibria, thereby preventing an accurate thermodynamic analysis of the self-assembly of **Agg II**.

Since **Agg IIc** does not participate in the solution equilibria, any quantitative transformation into the thermodynamically stable self-assembled structures must incorporate an energetic penalty for the rupture of the superstructures, making the therein “trapped” polymers of **Agg II** re-available to the solution-based processes. Therefore, the thermodynamic assessment derived from temperature-dependent UV-Vis spectroscopy overestimates the values of the thermodynamic parameters (Tables S1 and S2†). In contrast, the denaturation approach hinders the formation of superstructures due to the gradual increase in solvent polarity, which improves the solvation of the paraffinic side chains. Also, the constant mechanical agitation upon mixing the solutions after addition of the CHCl_3_ aliquots further helps to maintain the aggregates in solution.

The major impact of the fast kinetics of clustering on the overall equilibrium processes in solution is also reflected in the inability of the system to undergo living supramolecular polymerization. Upon addition of seeds of **Agg I** (obtained *via* sonication) to a solution of **Agg II**, no **Agg II** → **Agg I** conversion is observable. Instead, clustering of the kinetic species occurs on a faster time scale (Fig. S21†).

However, at this stage, it remains to be clarified why **Agg II** is never observed in any temperature-induced polymerization. The hidden character of this kinetic pathway can be rationalized by inspecting the plot of degree of aggregation (*α*_Agg_) against temperature for both polymorphs (Fig. 6b). The elongation temperature of **Agg II** derived from the heating process 
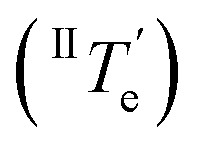
 is 343.6 K, whereas the critical temperature to be overcome for the cooling process of **Agg I**
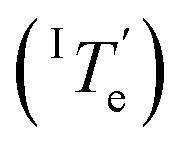
 lies much higher, around 360 K. Although the thermal hysteresis 
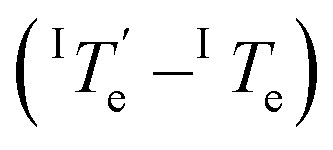
 for the thermodynamic pathway is slightly more pronounced as the cooling rate increases, ^I^*T*_e_ never falls below the elongation temperature necessary to initiate the polymerization of kinetic **Agg II** (^II^*T*_e_; Fig. S22†). Thus, in cooling-induced polymerization, the nuclei of thermodynamic **Agg I** will always elongate prior to formation of **Agg II** nuclei.

On the other hand, the cooperative character and the relative position of the melting curves in Fig. 6b disclose why kinetic **Agg II** undergoes a subsequent clustering process into **Agg IIc** at room temperature rather than evolving into the thermodynamic polymorph **Agg I**. In stark contrast to most kinetic pathways reported in the literature (isodesmic), the cooperative mechanism exhibited by **Agg II** enables high degrees of polymerization (*α*_Agg_ ≈ 1) in a very broad temperature range (approx. 288–313 K). Hence, the fraction of available monomers in this temperature range is so low, that nucleation of **Agg I** is inhibited. On the contrary, the clustering process does not require monomers but rather uses **Agg II** species as feedstock, explaining why this process is highly favourable under these conditions. Therefore, a transition to **Agg I** can only be possible when a sufficiently high concentration of monomers is present, which can be theoretically regulated by raising the temperature above 313 K. On this basis, the thermal annealing experiments were performed at 323 K (Fig. 6a) and 333 K (Fig. S18 and S19†), temperatures at which *α*_Agg_ of **Agg II** is lower than unity (between 0.9 and 0.7, dashed lines in Fig. 6b).

The direct, gradual transition from **Agg II** to **Agg I** without the intermediate appearance of the monomer spectra is also reflected in the speciation curves shown in Fig. 6c (for details, see ESI†). At both annealing temperatures (323 K and 333 K), the *α*_Agg_ value for **Agg I** is nearly unity. Under these conditions, any monomer that is released by the disassembly of **Agg II** is instantly converted to polymorph **Agg I**. Consequently, the monomer species cannot be observed in the spectra and there is a pseudo-isosbestic point (inset Fig. 6a, S18 and S19†), although the concentration-dependency clearly proves that the transformation is of competitive nature and, hence, proceeds *via* free monomer.

Under this assumption, the molar fraction of both **Agg I** and monomer *vs.* temperature could be derived from the heating curves (Fig. 6c). At temperatures below 313 K, **Agg II** is thermally inert. Raising the temperature decreases the population of **Agg II** at the expense of **Agg I**, which becomes the dominant species above approx. 338 K, where the curves intersect. At 
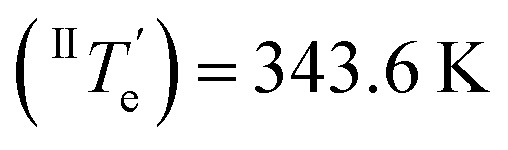
, **Agg II** is depleted and the molar fraction of **Agg I** reaches its maximum. Any subsequent temperature increase leads to disassembly of **Agg I**. These estimated trends perfectly agree with the experimentally observed UV-Vis heating experiments of **Agg II**, where **Agg I** could be identified before complete disassembly at high temperatures (Fig. 5b). Furthermore, plot 6c gives an estimate of the temperature window at which transformation of **Agg II** to **Agg I** is possible *via* thermal annealing (313–358 K, dashed grey lines). In fact, the **Agg II** → **Agg I** conversion could be confirmed at 313 K (the lower boundary of the temperature range; Fig. S20a†), further supporting the good correlation between the experimental results and the speciation plots.

## Conclusion and outlook

In summary, by thoroughly examining the self-assembly of a chiral Pd^II^ complex **1**, we have shown that hidden kinetic pathways can have dramatic consequences on supramolecular polymerization processes. Detailed spectroscopic studies at variable temperatures demonstrate that thermally controlled assembly protocols exclusively produce thermodynamically stable cooperative supramolecular polymers (**Agg I**) with no traces of any kinetic state. This can be explained by the lower *T*_e_ of the kinetic pathway compared to the thermodynamic product, a phenomenon that is in stark contrast to the typical behaviour of supramolecular polymers. On the other hand, solvophobic quenching, *i.e.* injecting the monomer species into a large volume of an aggregating solvent (MCH) allowed us to obtain a “hidden” kinetic polymerization pathway (**Agg II**) that is also cooperative in nature. This kinetic pathway evolves rapidly to clustered superstructures (**Agg IIc**) under preservation of the packing mode. Strikingly, once monomers have engaged into the kinetic pathway (**Agg II**), they are sequestered from the coupled polymerization equilibria in solution by the rapid clustering step, which prevents the system to redirect polymerization to the thermodynamic pathway, even in the presence of seeds. Such a drastic influence of a hidden kinetic pathway on the temporal evolution of two polymers is unprecedented. Our results demonstrate that the existence of hidden kinetic states can have a dramatic impact on the systems thermodynamics, enabling new aggregation pathways with potentially new materials and functional properties. Therefore, assuming that a given self-assembling system exists as a single thermodynamic product should be treated with care, unless a wide variety of experimental parameters and sample treatment methods have been tested.

## Conflicts of interest

There are no conflicts to declare.

## Supplementary Material

Supplementary information

## References

[cit1] Mattia E., Otto S. (2015). Nat. Nanotechnol..

[cit2] Grzybowski B. A., Huck W. T. S. (2016). Nat. Nanotechnol..

[cit3] Ogi S., Sugiyasu K., Manna S., Samitsu S., Takeuchi M. (2014). Nat. Chem..

[cit4] Dhiman S., Sarkar A., George S. J. (2018). RSC Adv..

[cit5] Jain A., Dhiman S., Dhayani A., Vemula P. K., George S. J. (2019). Nat. Commun..

[cit6] A de Greef T. F., Smulders M. M. J., Wolffs M., Schenning A. P. H. J., Sijbesma R. P., Meijer E. W. (2009). Chem. Rev..

[cit7] Rest C., Kandanelli R., Fernández G. (2015). Chem. Soc. Rev..

[cit8] Matern J., Dorca Y., Sánchez L., Fernández G. (2019). Angew. Chem., Int. Ed..

[cit9] Korevaar P. A., George S. J., Markvoort A. J., Smulders M. M. J., Hilbers P. A. J., Schenning A. P. H. J., de Greef T. F. A., Meijer E. W. (2012). Nature.

[cit10] Valera J. S., Gómez R., Sánchez L. (2017). Small.

[cit11] Ogi S., Fukui T., Jue M. L., Takeuchi M., Sugiyasu K. (2014). Angew. Chem., Int. Ed..

[cit12] Wagner W., Wehner M., Stepanenko V., Ogi S., Würthner F. (2017). Angew. Chem., Int. Ed..

[cit13] Wang H., Zhang Y., Chen Y., Pan H., Ren X., Chen Z. (2020). Angew. Chem., Int. Ed..

[cit14] Chen Z., Liu Y., Wagner W., Stepanenko V., Ren X., Ogi S., Würthner F. (2017). Angew. Chem., Int. Ed..

[cit15] Ogi S., Stepanenko V., Thein J., Würthner F. (2016). J. Am. Chem. Soc..

[cit16] Greciano E. E., Matarranz B., Sánchez L. (2018). Angew. Chem., Int. Ed..

[cit17] Aratsu K., Takeya R., Pauw B. R., Hollamby M. J., Kitamoto Y., Shimizu N., Takagi H., Haruki R., Adachi S.-I., Yagai S. (2020). Nat. Commun..

[cit18] Fukui T., Sasaki N., Takeuchi M., Sugiyasu K. (2019). Chem. Sci..

[cit19] Langenstroer A., Kartha K. K., Dorca Y., Droste J., Stepanenko V., Albuquerque R. Q., Hansen M. R., Sánchez L., Fernández G. (2019). J. Am. Chem. Soc..

[cit20] Suzuki A., Aratsu K., Datta S., Shimizu N., Takagi H., Haruki R., Adachi S.-I., Hollamby M., Silly F., Yagai S. (2019). J.J.
Am. Chem. Soc.Am. Chem. Soc..

[cit21] Haedler A. T., Meskers S. C. J., Zha R. H., Kivala M., Schmidt H.-W., Meijer E. W. (2016). J. Am. Chem. Soc..

[cit22] Herkert L., Droste J., Kartha K. K., Korevaar P. A., de Greef T. F. A., Hansen M. R., Fernández G. (2019). Angew. Chem., Int. Ed..

[cit23] Yagai S., Yamauchi M., Kobayashi A., Karatsu T., Kitamura A., Ohba T., Kikkawa Y. (2012). J. Am. Chem. Soc..

[cit24] Hifsudheen M., Mishra R. K., Vedhanarayanan B., Praveen V. K., Ajayaghosh A. (2017). Angew. Chem., Int. Ed..

[cit25] Markiewicz G., Smulders M. M. J., Stefankiewicz A. R. (2019). Adv. Sci..

[cit26] Ogi S., Stepanenko V., Sugiyasu K., Takeuchi M., Würthner F. (2015). J. Am. Chem. Soc..

[cit27] Liu Y., Gong Y., Guo Y., Xiong W., Zhang Y., Zhao J., Che Y., Manners I. (2019). Chem.–Eur. J..

[cit28] Langenstroer A., Dorca Y., Kartha K. K., Mayoral M. J., Stepanenko V., Fernández G., Sánchez L. (2018). Macromol. Rapid Commun..

[cit29] Wan Q., To W.-P., Chang X., Che C.-M. (2020). Chem.

[cit30] Zou C., Lin J., Suo S., Xie M., Chang X., Lu W. (2018). Chem. Commun..

[cit31] Chow P.-K., To W.-P., Low K.-H., Che C.-M. (2014). Chem.–Asian J..

[cit32] Yam V. W.-W., Au V. K.-M., Leung S. Y.-L. (2015). Chem. Rev..

[cit33] Wan Q., To W.-P., Yang C., Che C.-M. (2018). Angew. Chem., Int. Ed..

[cit34] Ghosh G., Ghosh T., Fernández G. (2020). ChemPlusChem.

[cit35] Aliprandi A., Mauro M., de Cola L. (2016). Nat. Chem..

[cit36] Xiao X.-S., Lu W., Che C.-M. (2014). Chem. Sci..

[cit37] Chen Y., Che C.-M., Lu W. (2015). Chem. Commun..

[cit38] Allampally N. K., Mayoral M. J., Chansai S., Lagunas M. C., Hardacre C., Stepanenko V., Albuquerque R. Q., Fernández G. (2016). Chem.–Eur. J..

[cit39] Chen M., Wei C., Wu X., Khan M., Huang N., Zhang G., Li L. (2015). Chem.–Eur. J..

[cit40] Wolffs M., George S. J., Tomović ž., Meskers S. C. J., Schenning A. P. H. J., Meijer E. W. (2007). Angew. Chem., Int.Angew. Chem., Int.
Ed.Ed..

[cit41] Buendía J., Calbo J., Ortí E., Sánchez L. (2017). Small.

[cit42] Bäumer N., Kartha K. K., Allampally N. K., Yagai S., Albuquerque R. Q., Fernández G. (2019). Angew. Chem., Int. Ed..

[cit43] Kartha K. K., Allampally N. K., Politi A. T., Prabhu D. D., Ouchi H., Albuquerque R. Q., Yagai S., Fernández G. (2019). Chem. Sci..

[cit44] Kartha K. K., Allampally N. K., Yagai S., Albuquerque R. Q., Fernández G. (2019). Chem.–Eur. J..

[cit45] Rest C., Mayoral M. J., Fucke K., Schellheimer J., Stepanenko V., Fernández G. (2014). Angew. Chem., Int. Ed..

[cit46] Wehner M., Röhr M. I. S., Bühler M., Stepanenko V., Wagner W., Würthner F. (2019). J. Am. Chem. Soc..

[cit47] Shirakawa M., Kawano S.-I., Fujita N., Sada K., Shinkai S. (2003). J. Org. Chem..

[cit48] Rest C., Martin A., Stepanenko V., Allampally N. K., Schmidt D., Fernández G. (2014). Chem. Commun..

[cit49] Markvoort A. J., ten Eikelder H. M. M., Hilbers P. A. J., de Greef T. F. A., Meijer E. W. (2011). Nat. Commun..

[cit50] Korevaar P. A., Schaefer C., de Greef T. F. A., Meijer E. W. (2012). J. Am. Chem. Soc..

[cit51] Cruz-Cabeza A. J., Reutzel-Edens S. M., Bernstein J. (2015). Chem. Soc. Rev..

[cit52] Bernstein J., Polymorphism in Molecular Crystals, Oxford University Press, Oxford, 2007.

[cit53] Gale P. A. and Steed J. W., Supramolecular chemistry. From molecules to nanomaterials, Wiley, Weinheim, 2006.

[cit54] Liu Y., Zhang Y., Fennel F., Wagner W., Würthner F., Chen Y., Chen Z. (2018). Chem.–Eur. J..

