# Supplementary material for: Consequences of hidden kinetic pathways on supramolecular polymerization
Source: Chem Sci. 2020 Jun 2;11(26):6780–8. doi: 10.1039/d0sc02115f (PMC7450716; doi:10.1039/d0sc02115f)
Supplement: Supplementary file 1 [file SC-011-D0SC02115F-s001.pdf]

Electronic Supplementary Material (ESI) for Chemical Science.
This journal is © The Royal Society of Chemistry 2020

Supporting Information

Consequences of Hidden Kinetic Pathways on Supramolecular Polymerization

Jonas Matern[a], Kalathil K. Kartha[a], Luis Sánchez[b] and Gustavo Fernández*[a]

[a] J. Matern, Dr. Kalathil K. Kartha, Prof. Dr. G. Fernández
Organisch-Chemisches Institut
Westfälische Wilhelms-Universität Münster
Corrensstraße 40, 48149 Münster (Germany)
E-mail: fernandg@uni-muenster.de

[b] Prof. Dr. L. Sánchez
Departamento de Química Orgánica, Facultad de Ciencias Químicas
Universidad Complutense de Madrid,
Ciudad Universitaria s/n, 28040 Madrid (Spain)

Summary of Contents

1. Nucleation-Elongation Model for cooperative Supramolecular Polymerizations 2
2. Denaturation Model for Cooperative Supramolecular Polymerizations 3
3. Thermodynamic Parameters 4
4. Supplementary Figures 5
5. Calculation of the molar fraction of Agg I and Monomer *vs.* Temperature 17
6. Experimental Part 18
   6.1. Materials and Methods 18
   6.2. Synthesis and Characterization 20

1. Nucleation-Elongation Model for cooperative Supramolecular Polymerizations

The equilibrium between the monomeric and supramolecular polymer species can be described in a cooperative process with the *Nucleation-Elongation model* which was developed by Ten Eikelder, Markvoort and Meijer[1,2]. This model is used to describe the aggregation of **1** which exhibits a non-sigmoidal cooling curve as shown in temperature dependent UV-Vis experiments. The model extends nucleation-elongation based equilibrium models for growth of supramolecular homopolymers to the case of two monomer and aggregate types and can be applied to symmetric supramolecular copolymerizations, as well as to the more general case of nonsymmetric supramolecular copolymerizations.

In a cooperative process the polymerization occurs *via* two steps: a nucleation step, where a nucleus, which is assumed to have a size of 2 molecules, is formed and a following elongation step. The values T_e, $\Delta H°_{nucl}$, $\Delta H°$ and $\Delta S°$ can be found by a non-linear least-square analysis of the experimental melting curves. The equilibrium constants associated with the nucleation and elongation phases can be calculated using the following equations:

Nucleation step: $K_n = e^{\left(\frac{-\left(\left(\Delta H^o - \Delta H_{NP}^o\right) - T\Delta S^o\right)}{RT}\right)}$

Elongation step: $K = e^{\left(\frac{-\left(\Delta H^o - T\Delta S^o\right)}{RT}\right)}$

Furthermore, the cooperativity factor (σ) is given by:

$$\sigma = \frac{K_n}{K_e} = e^{\left(\frac{\Delta H_{NP}^o}{RT}\right)}$$

[1] H. M. M. Ten Eikelder, A. J. Markwoort, T. F. A. De Greef, and P. A. J. Hilbers, *J. Phys. Chem. B* **2012**, *116*, 5291-5301.
[2] A. J. Maarkvort, H. M. M. Ten Eikelder, P. J. J. Hilbers, T. F. A. De Greef and E. W. Meijer, *Nat. Commun.* **2011**, *2*, 509-517.

2. Denaturation Model for Cooperative Supramolecular Polymerizations[3]

The denaturation model is based on the concentration-dependent supramolecular polymerization equilibrium model by Goldstein,[4] where the polymerization is described as a sequence of monomer addition equilibria.

$[P_n] = K_n[P_{n-1}][X]$

$[P_{n+1}] = K_e[P_n][X]$

$[P_i] = K_e[P_{i-1}][X]$

For the cooperative model $K_n < K_e$ and for isodesmic process $K_n = K_e$. The concentration for each species P_i is given by $[P_i] = K^{i-1}_n[X]^i$ for $i \leq n$ and $[P_i] = K^{i-n}_eK^{n-1}_n[X]^i$ for $i > n$.

The dimensionless mass balance is obtained by inserting the dimensionless concentration $p_i = K_e[P_i]$, the monomer concentration $x = K_e[X]$ and the concentration of each species P_i (for $i \leq n$) : $p_i = \sigma^{i-1}x^i$ and for $i > n : p_i = \sigma^{n-1}x^i$):

$$x_{tot} = \sigma^{-1}\sum_{i=1}^{n} i(\sigma x)^i + \sigma^{n-i}\sum_{i=n+1}^{\infty} ix^i$$

Both sums are evaluated by using standard expressions for converging series:

$$x_{tot} = \left(\frac{(\sigma x)^{n+1}(n\sigma x - n - 1)}{(\sigma x - 1)^2} + \frac{\sigma x}{(\sigma x - 1)^2}\right) - \sigma^{n-1}\left(\frac{x^{n+1}(nx - n - 1)}{(x - 1)^2}\right)$$

With $x_{tot} = c_{tot}K_e$ and c_{tot}: total monomer concentration.

The sum solved by standard numerical methods (Matlabfzerosolver) yields the dimensionless monomer concentration x. Considering that every species with $i > 1$ is defined as aggregate, the degree of aggregation results in:

$$\alpha_{Agg} = \frac{x_{tot} - x}{x_{tot}}$$

Via $K_e = exp(-\frac{\Delta G^0}{RT})$, the denaturation curves can be obtained with *f* defined as volume fraction of good solvent:

$$\Delta G^{0,\prime} = \Delta G^0 + m\,f$$

It is assumed that the cooperativity factor σ is independent on the volume fraction and the *m* value for the elongation regime equals the *m* value for nucleation.

The denaturation data needs to be transformed into the normalized degree of aggregation, if fitted to the supramolecular polymerization equilibrium model:

---

[3] P. A. Korevaar, C. Schaefer, T. F. A. de Greef, E. W. Meijer, *J. Am. Chem. Soc.*, 2012, **134**, 13482–13491.

[4] R. F. Goldstein and L. Stryer *Biophys. J.*, 1986, **50**, 583-599.

$$(f) = \frac{A(f) - A(f=0)}{A(f=1) - A(f=0)}$$

The optimization of the four needed parameters (ΔG_0, m, σ and p) to fit the equilibrium model to the experimental data (normalized degree *vs. f*) is done by the non-linear least-squares analysis using Matlab (lsqnonlinsolver). The data is then fitted with the non-linear least squared regression (Levenberg-Marquardt algorithm).

3. Thermodynamic Parameters

The thermodynamic Parameters (**Table S1+S2**) were obtained fitting respective experimental data to the nucleation-elongation model or the denaturation model. VT fits were obtained by simultaneously fitting multiple concentrations (**Agg I**: 3, 4 ,5 µM; **Agg II**: 2.5, 5, 7.5, 10 µM).

Table S1: Thermodynamic parameters obtained for **Agg I**

	c[a]	ΔH_0 [b]	ΔS_0 [c]	ΔH_{NP} [b]	T_{ed}	ΔG_{298} [b]	K_e [e]	K_{nucl} [e]	σ
**EQ model VT-fit**	3, 4, 5	-106.8	-190.0	-24.8	361.0 (3 µM)<br>363.9 (4 µM)<br>366.2 (5 µM)	-50.1	3.3×10^5	87.3	2.6×10^5
**Denat. fit**	5					-51.5			6.6×10^2

[a] in µM; [b] in in kJmol^{-1}; [c] in JK^{-1}mol^{-1}; [d] in K, the values in parentheses correspond to the utilized concentration; [e] in M^{-1}

Table S2: Thermodynamic parameters obtained for **Agg II**

	c[a]	ΔH_0 [b]	ΔS_0 [c]	ΔH_{NP} [b]	T_{ed}	ΔG_{298} [b]	K_e [e]	K_{nucl} [e]	σ
**EQ model VT-fit**	2.5, 5, 7.5, 10	-286.2	743.0	-8.9	336.5 (5 µM)<br>338.8 (7.5 µM)<br>340.1 (10 µM)<br>341.1 (12.5 µM)	-64.6	4.0×10^5	1.7×10^4	4.2×10^{-2}
**Denat. fit**	5					-41.6			1.1×10^{-2}

[a] in µM; [b] in in kJmol^{-1}; [c] in JK^{-1}mol^{-1}; [d] in K, the value in parentheses correspond to the utilized concentration; [e] in M^{-1}

4. Supplementary Figures

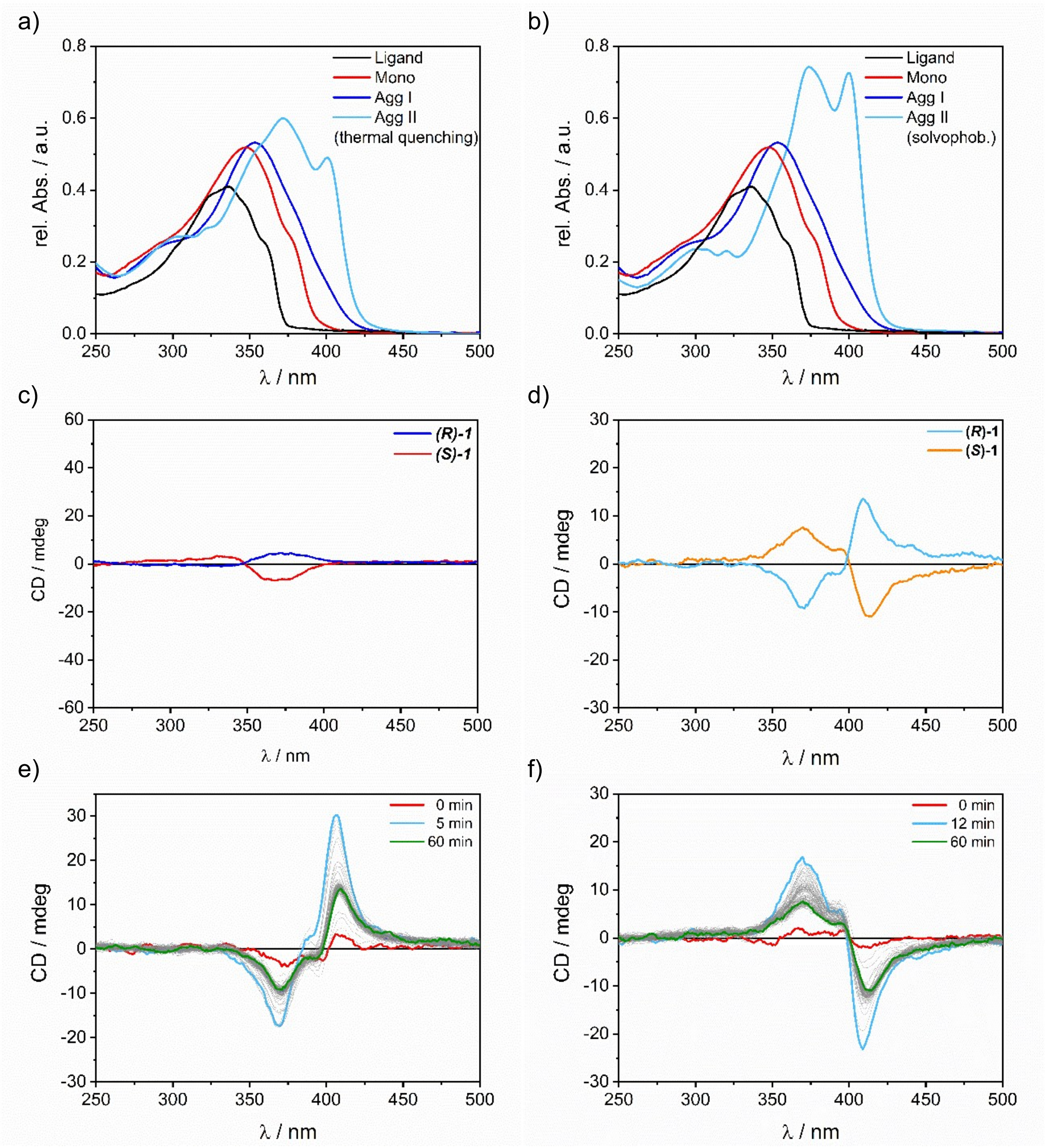

Figure S1: UV-Vis spectra of free ligand (black, c = 1 x 10^{-5} M) monomer (red, c = 5 x 10^{-6} M), **Agg I** (blue, c = 5 x 10^{-6} M) and **Agg II** (cyan, c = 5 x 10^{-6} M) prepared by a) thermal quenching, b) solvophobic quenching. CD spectra of **Agg I** (c) and **Agg II** (d) for (*R*)- and (*S*)-enantiomer (c = 5 x 10^{-6} M, for all α = 1), showing that both enantiomers form identical aggregates. Time dependent CD-spectra of **Agg II** for **(*R*)-1** (e) and **(*S*)-1** (f).

The slight difference in the intensity of the spectra of **Agg II** (d) originates from the high sensitivity of the CD measurements towards further growth/clustering of **Agg II** which results in a strong LD signal affecting the CD intensities (see Fig.S5). As these processes occur very quickly after quenching, the intensity depends strongly on the exact point in time when the spectra are measured (see time dependent spectra (e)+(f)). Nevertheless, the spectral features are almost identical, which proves that both enantiomers form the same supramolecular polymers, but with inverse CD signals.

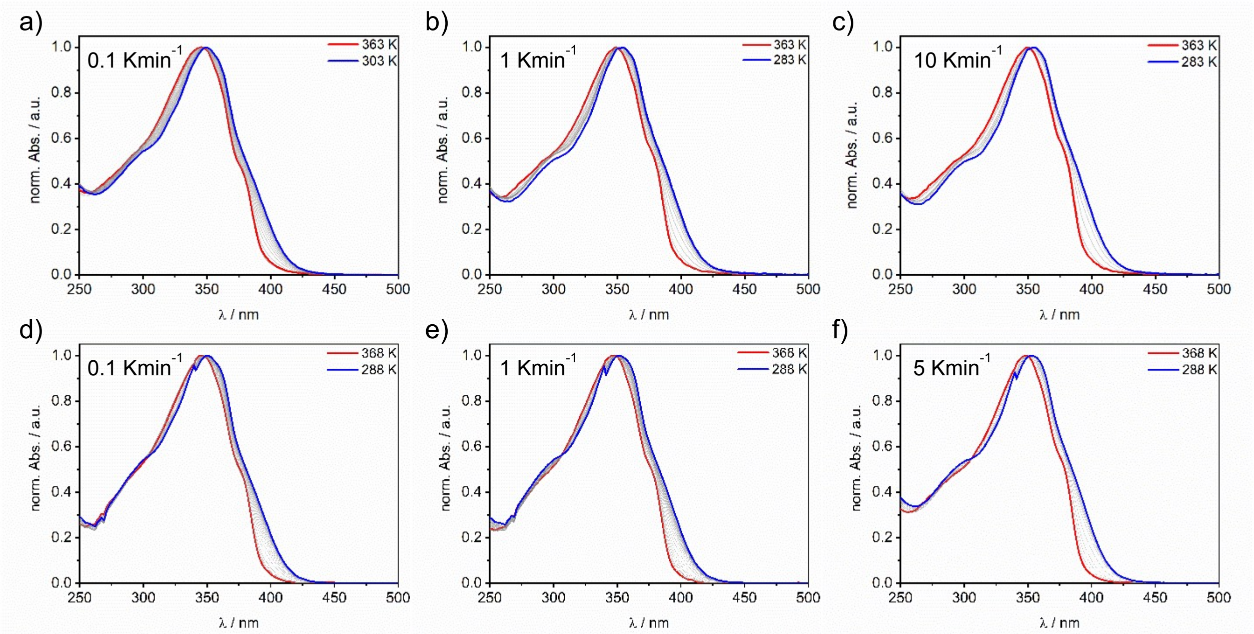

Figure S2: VT UV-Vis spectra obtained upon cooling solutions of (*R*)-**1** (a-c) and (*S*)-**1** (d-f) at different cooling rates (c = 5 x 10^{-6} M).

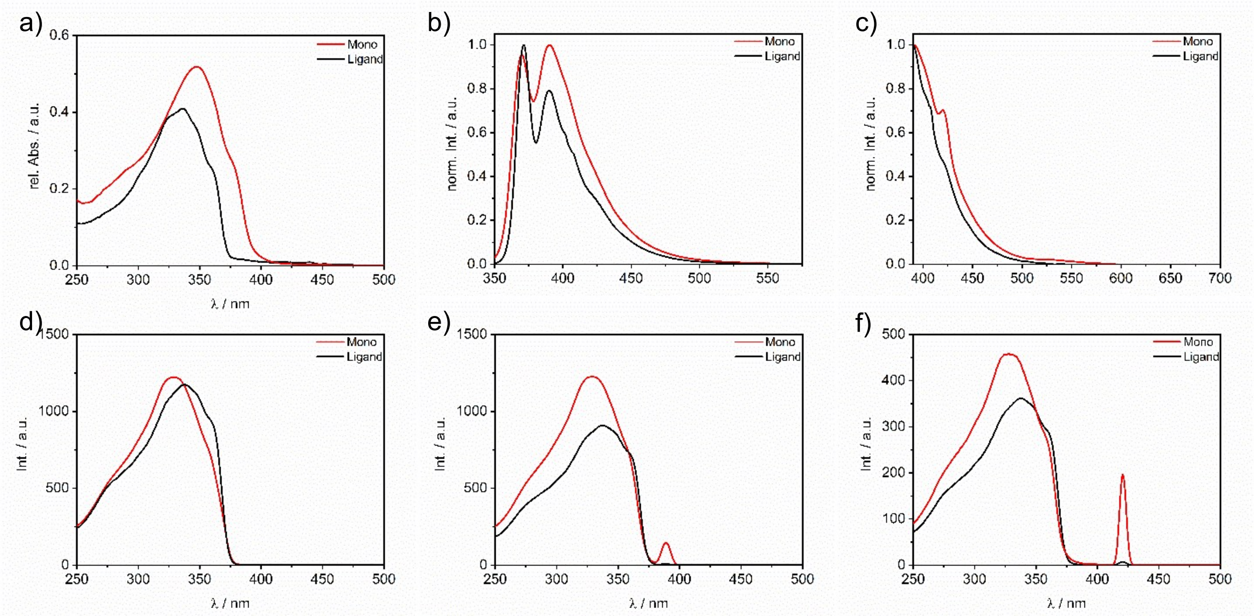

Figure S3: Top panel: absorption (a) and emission spectra (b: λ_{exc} = 321 nm ,c: λ_{exc} = 375 nm) of free (*R*)-chiral ligand (c = 1 x 10^{-5} M, black) and monomeric complex **(*R*)-1** (c = 1 x 10^{-5} M, 368 K, red) in MCH. Bottom panel: corresponding excitation spectra recorded at the emission maxima (d: λ_{em} = 371 nm, e: λ_{exc} = 390 nm and f: λ_{exc} = 422 nm).

The red-shift and loss of vibronic fine structure of the absorption of the complex with respect to the free ligand and the similarity of the fluorescence spectra of both compounds, regardless of the excitation wavelength, indicate that the absorption of the complex originates from ligand-centered ^{1}IL ($\pi \rightarrow \pi^*$) transitions with possible ^{1}MLCT contributions. The similarity between the excitation spectra of complex and ligand confirms the contributions of ligand centered transitions. Excitation at 375 nm where the

complex absorbs strongly, but the ligand only poorly, again yields very similar fluorescence spectra. This indicates that the absorption of the complex at this wavelength does not solely originate from the IL transitions which exhibit fluorescence, but include non-fluorescent MLCT contributions. The corresponding excitation spectra recorded at 422 nm (f) confirm this assumption.

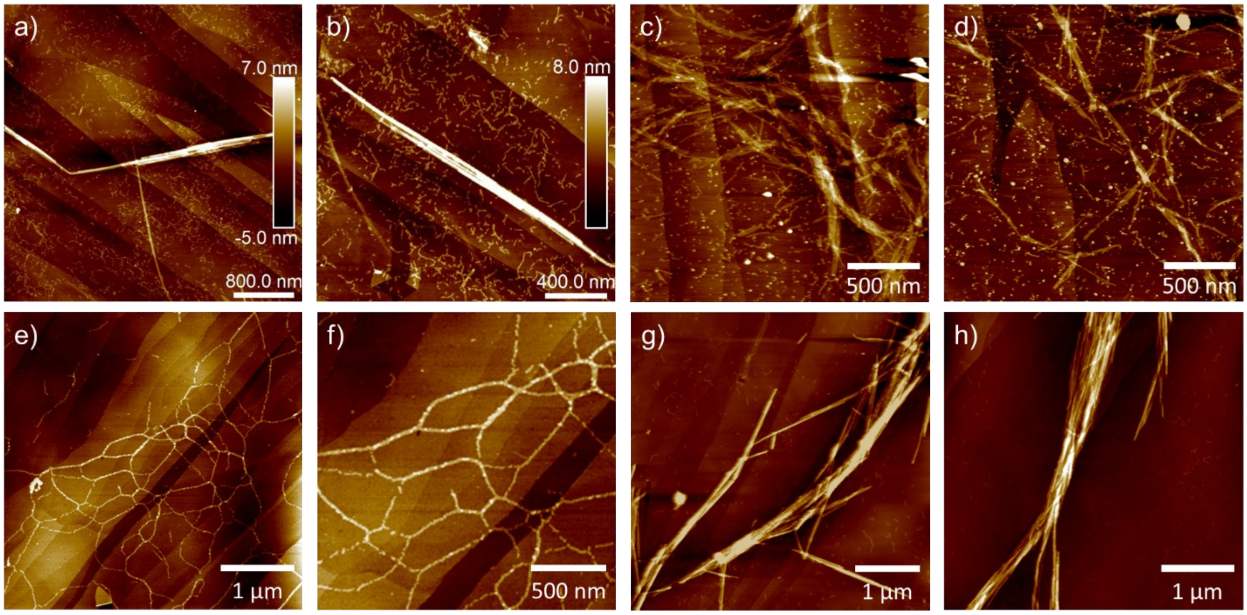

Figure S4: Top panel (a-d): AFM images of mixtures of both aggregates of (*R*)-**1** obtained upon thermal quenching (small structures: **Agg I**, big structures: **Agg II**). Bottom panel: additional AFM images of **Agg I** (e+f) and **Agg II/IIc** (g+h).

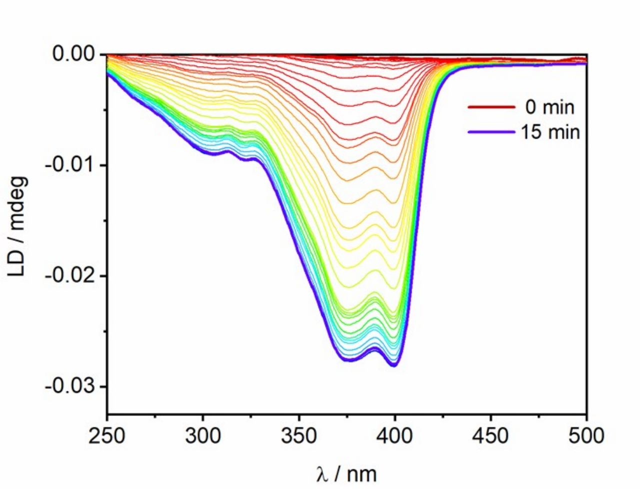

Figure S5: Linear dichroism of (*R*)-**1** evolving after the formation of **Agg II** (c = 5 x 10^{-6} M).

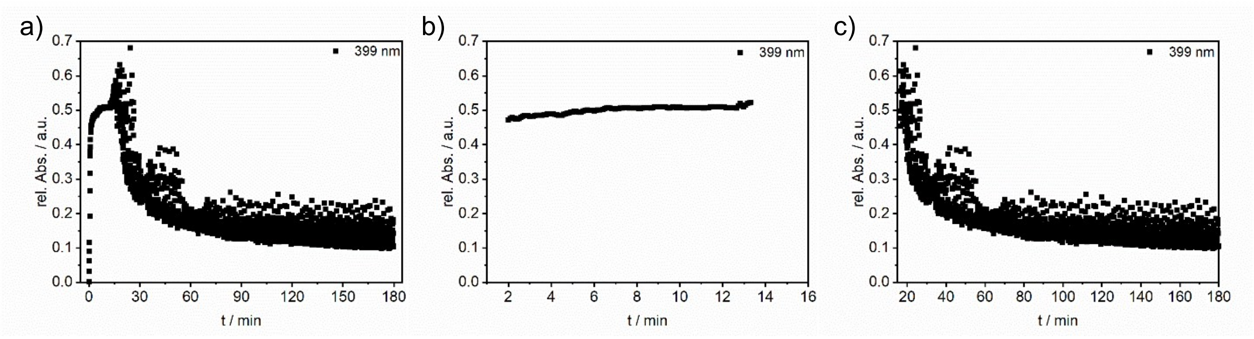

Figure S6: a) Development of the absorbance at 399 nm upon formation of **Agg II** over a time span of 180 min (c = 5 x 10^{-6} M). b) Zoom-in of the steady-state regime (2-15 min) corresponding to fully assembled **Agg II** and the time span where clustering to **Agg IIc** takes place (c).

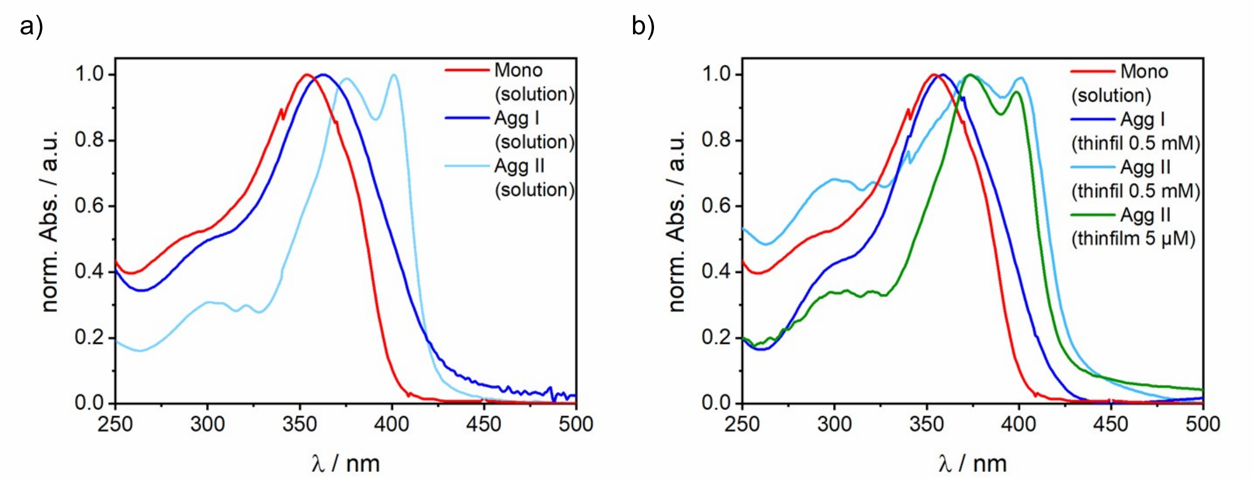

Figure S7: UV-Vis spectra of solutions (a) and thin films (b) of (R)-**1** showing the equivalence of in-solution and solid-state structures. Conditions: d = 0.1 mm, $CHCl_3$ for monomer (c = 0.5 x 10^{-3} M), MCH + 10% $CHCl_3$ for **Agg I**, MCH + 1 % $CHCl_3$ for **Agg II**.

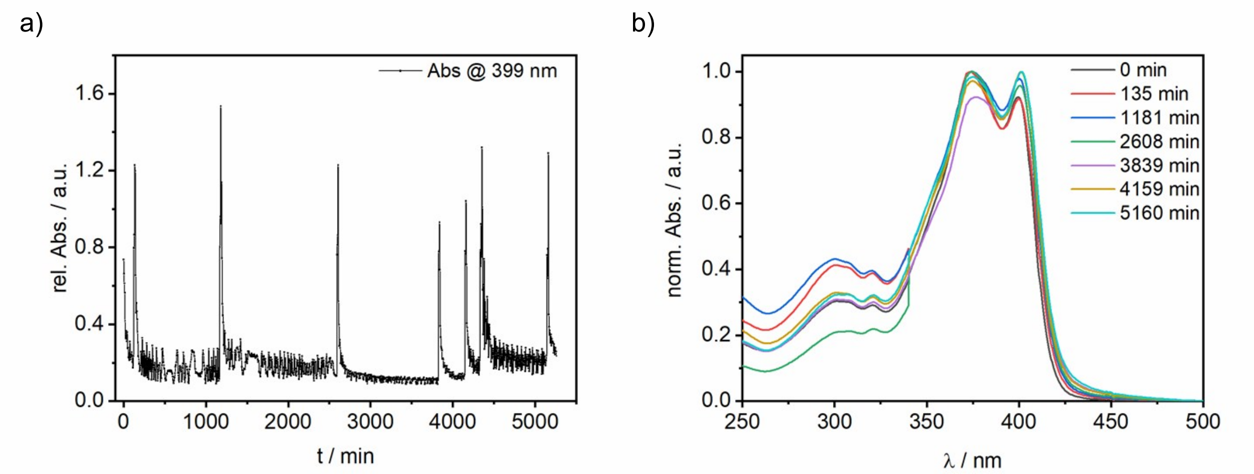

Figure S8: a) Repeated cycles of clustering over time and dissolution of the superstructures by shaking, visualized at 399 nm, showing the reversibility of the hierarchical step (c = 5 x 10^{-6} M). b) Corresponding UV-Vis spectra recorded after shaking (at peaks in a).

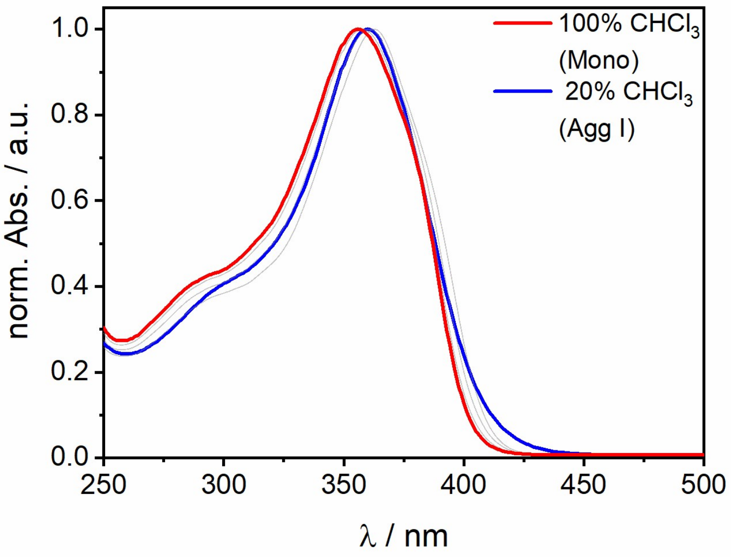

Figure S9: UV-Vis spectra of (*R*)-**1** recorded of the solutions used for NMR spectroscopic studies to determine the packing of **Agg I** upon varying the MCH-d_{14} volume fraction of the solution (c = 2 x 10^{-3} M).

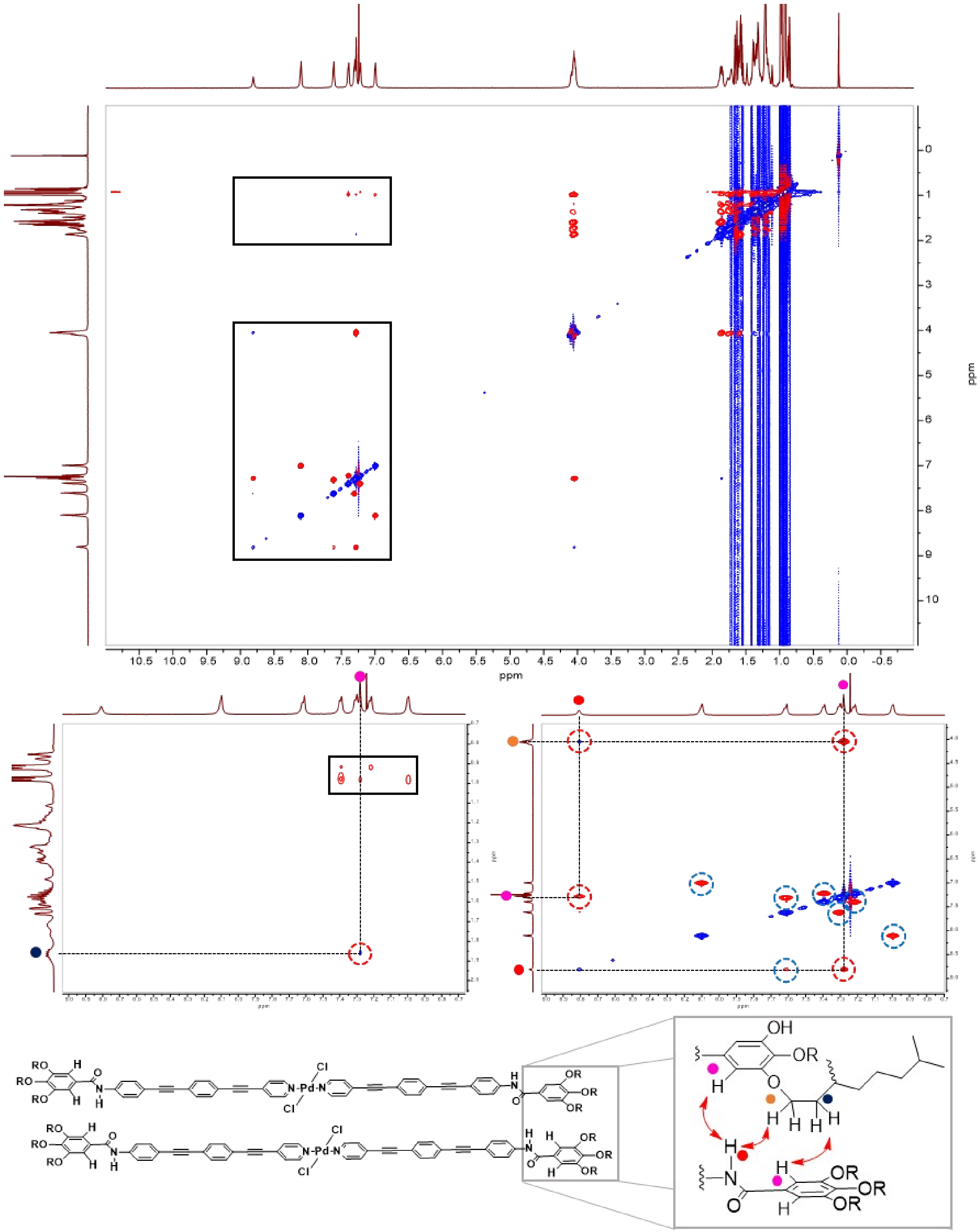

Figure S10: 2D ROESY NMR spectrum of (*R*)-**1** (**Agg I**, $c = 1 \times 10^{-2}$ M, 1:1 $CDCl_3$:MCH-d_{14}) and zoomed-in regions showing the correlation signals in the aliphatic and aromatic region, respectively.

The lack of through-space couplings of protons of the peripheral alkoxy chains with the aromatic core can be attributed to a parallel arrangement of the chromophores. Only the second CH_2 group of the solubilizing chains shows a weak coupling with the gallic C-H proton, which is possible in the proposed packing. The other cross peaks (black box) arise from interactions with the MCH-d_{14} solvent. In the aromatic region, a multitude of cross peaks is observed, however most of them originate from intramolecular couplings (blue circles). The through-space contacts are marked with red circles and their origin can be extracted from the schematic packing shown below the spectra.

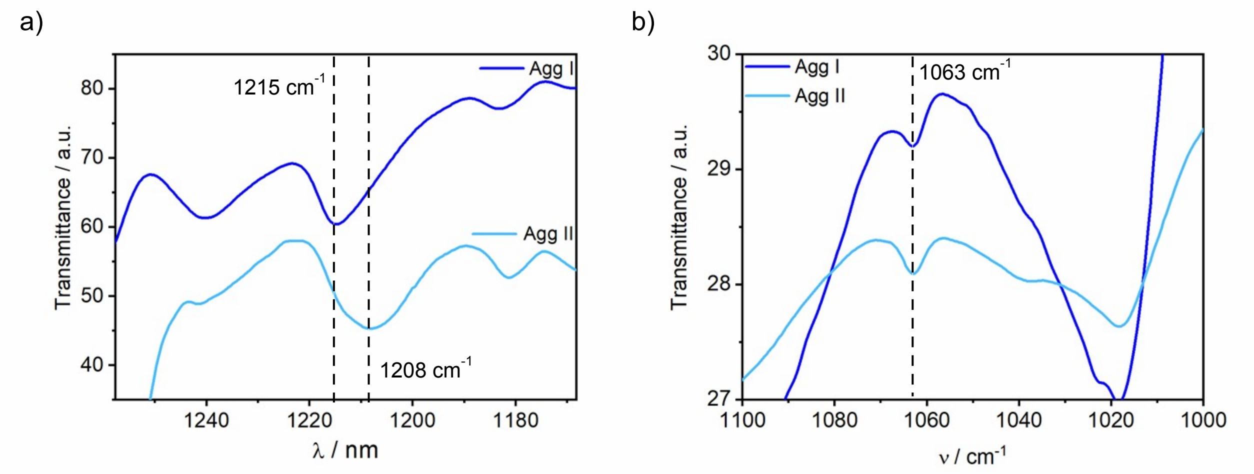

Figure S11: Partial thin-film FT-IR spectra of both aggregates of (*R*)-**1** in the spectral region characteristic of a) the C_{aryl}-OR band (1260-1160 cm^{-1}) and b) the *meta*-substituted ring vibration (1100-1000 cm^{-1}).

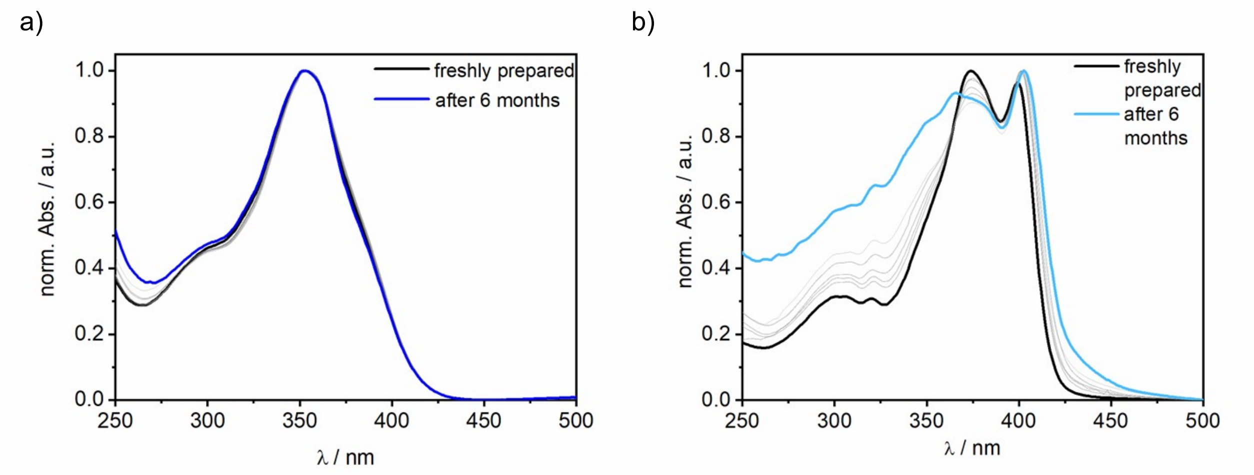

Figure S12: Time-dependent evolution of both **Agg I** (a) and **Agg II** (b) of (*R*)-**1** over a time span of six months (c = 5 x 10^{-6} M).

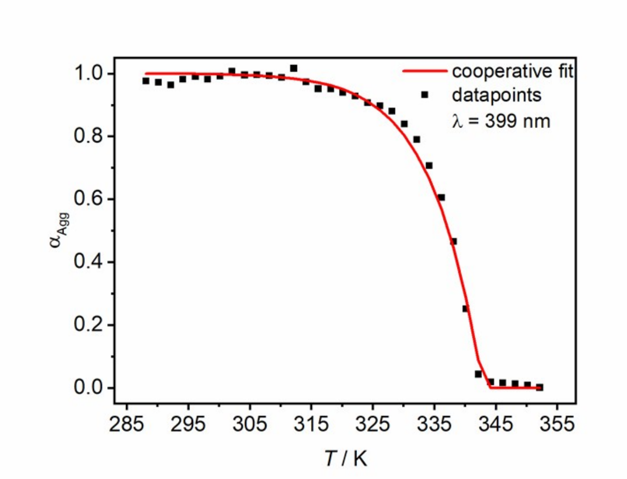

Figure S13: Experimental data points extracted for the disassembly of **Agg II** (c = 5 x 10^{-6} M) at λ = 399 nm and fit to the nucleation-elongation model showing the cooperative character of the transition.

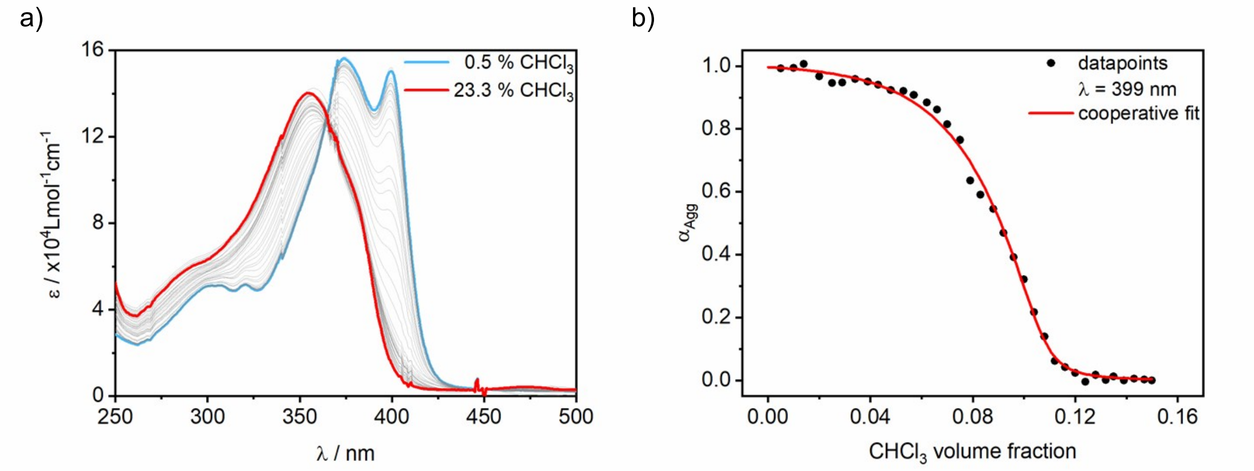

Figure S14: a) Solvent-dependent UV-Vis studies of (*R*)-**1** (**Agg II**) monitoring the disassembly *via* an increase in the volume fraction of good solvent at a constant concentration (c = 5 x 10^{-6} M) and corresponding cooperative fit using the denaturation model (b).

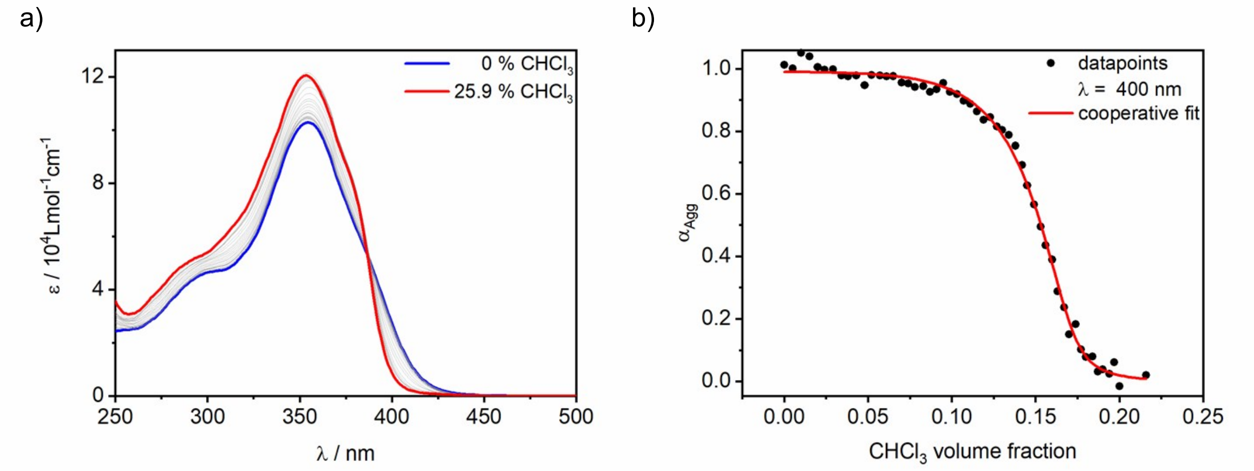

Figure S15: a) Solvent-dependent UV-Vis studies of (*R*)-**1** (**Agg I**) monitoring the disassembly *via* an increase in the volume fraction good solvent at a constant concentration (c = 5 x 10^{-6} M) and corresponding cooperative fit using the denaturation model (b).

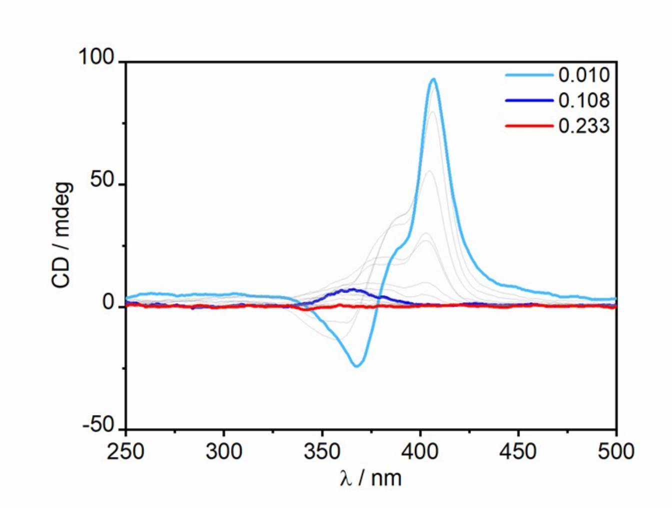

Figure S16: Solvent-dependent CD studies of (*R*)-**1** (**Agg II**) monitoring the disassembly *via* an increase in the volume fraction of good solvent at a constant concentration (c = 5 x 10^{-6} M) showing the intermediate formation of **Agg I** (blue spectrum).

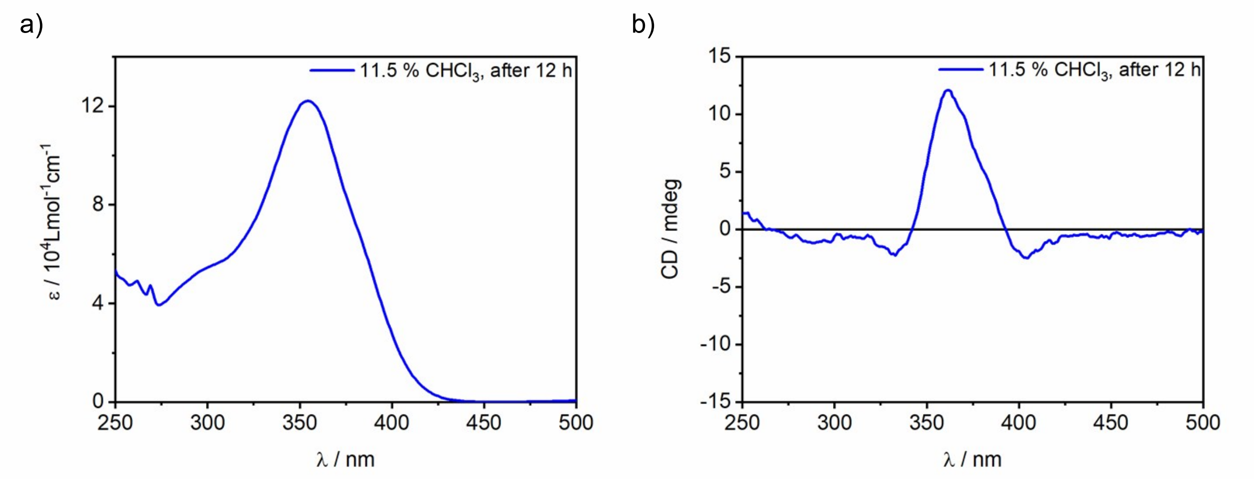

Figure S17: UV-Vis (a) and CD (b) spectrum of (*R*)-**1** showing the characteristic features of **Agg I** which was obtained during disassembly of **Agg II** at a chloroform volume fraction of 11.5 % (*c* = 5 x 10^{-6} M). The spectra were recorded 12 hours after addition of 11.5 % v/v chloroform to the MCH solution of **Agg II**, showing that **Agg I** is a persistent intermediate in the disassembly process of **Agg II** rather than a transient species.

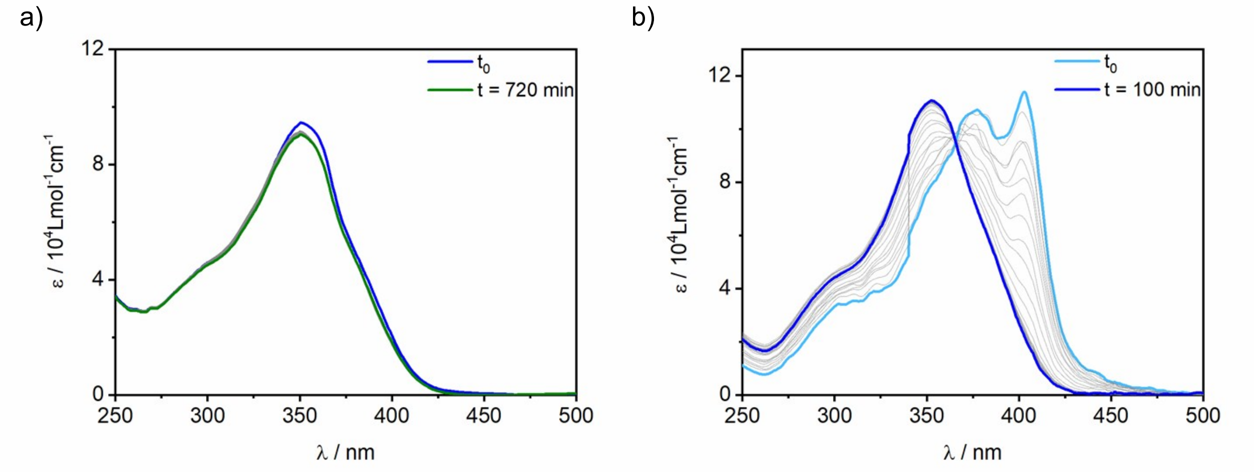

Figure S18: Time-dependent evolution of the UV-Vis spectra of (*R*)-**1** (**Agg I** (a) and **Agg II** (b)) upon annealing solutions (c = 5 x 10^{-6} M) of both polymorphs at 333 K. In the case of **Agg I** no changes occur, whereas **Agg II** transforms to **Agg I**.

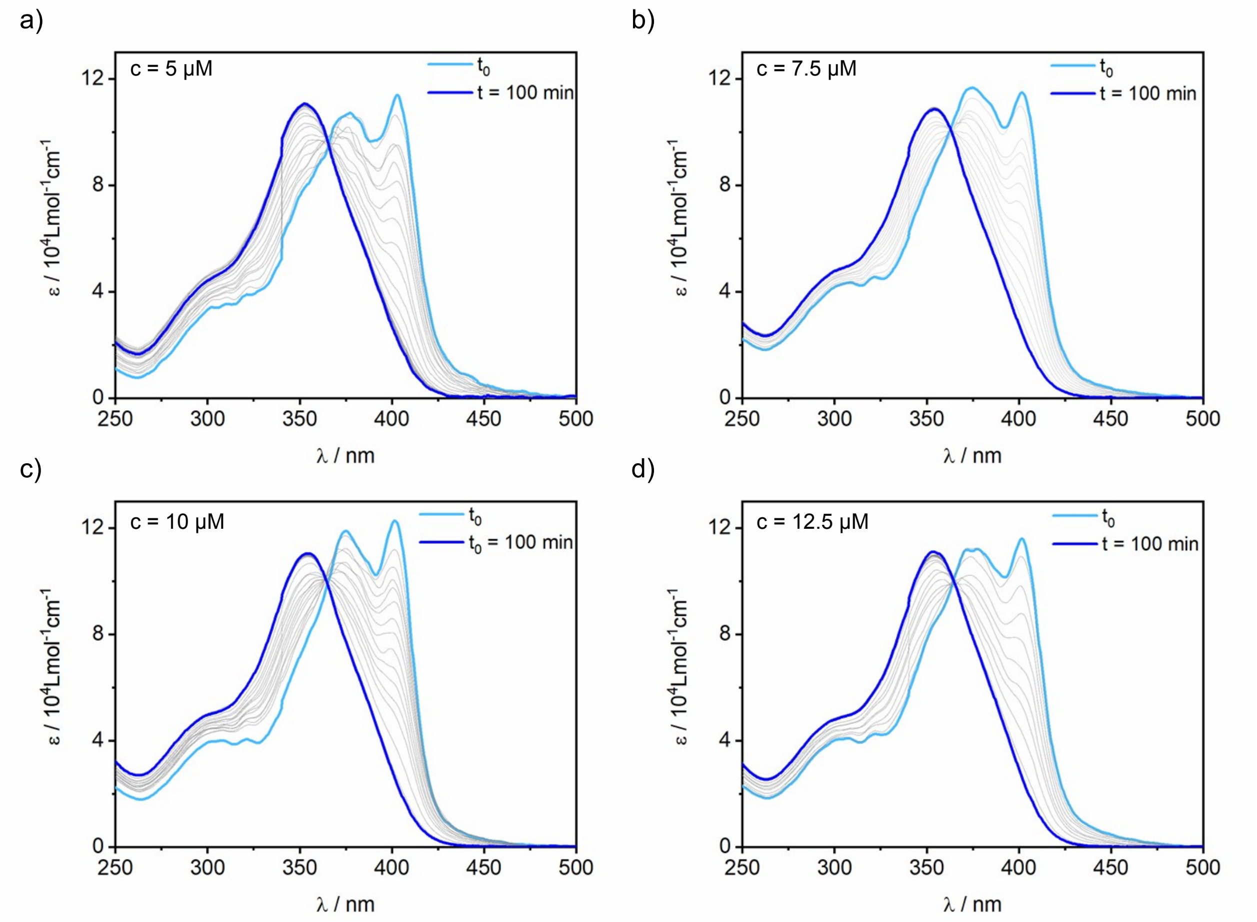

Figure S19: UV-Vis spectra of (*R*)-**1** recorded upon annealing solutions of freshly prepared **Agg II** at 333 K at different concentrations.

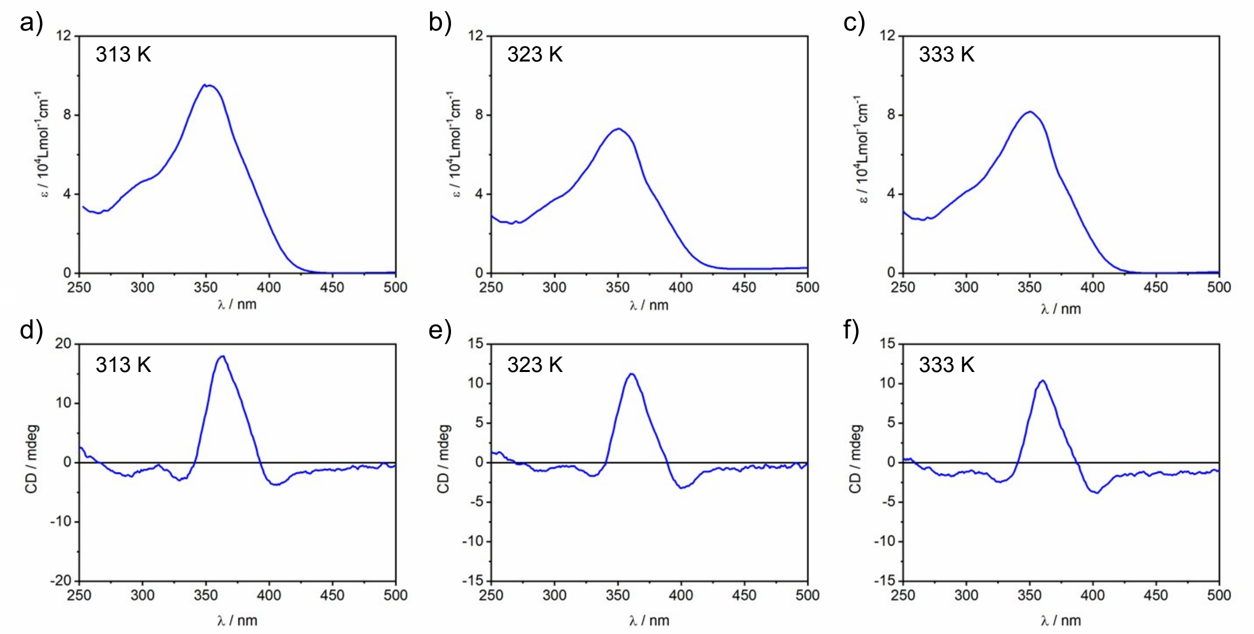

Figure S20: UV-Vis (a,b,c) and CD spectra (d,e,f) of (*R*)-**1** obtained after annealing solutions of freshly prepared **Agg II** at a,d) 313 K, b,e) 323 K and c,f) 333 K, showing the conversion to **Agg I** ($c = 5 \times 10^{-6}$ M).

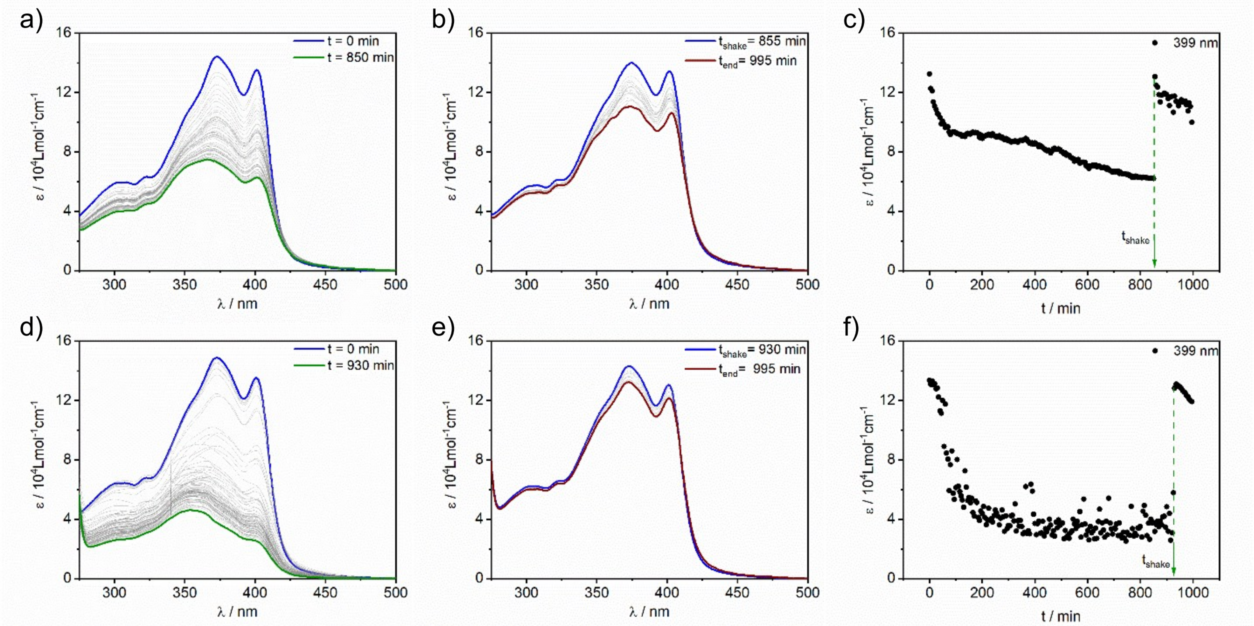

Figure S21: UV-Vis spectra of (*R*)-**1** recorded upon probing seeded supramolecular polymerization (ratio **Agg I$_{seed}$**:**Agg II** 1:1, $c = 5 \times 10^{-6}$ M) stirring at 1000 rpm (a,b,c) or without stirring (d,e,f). c,f) Plot of absorbance at 399 nm *vs.* time showing a rapid decay of absorbance. The results show that the supramolecular polymerization of **1** cannot proceed in a living manner. The spectral changes can be attributed to a lowering of the concentration of **Agg II** in solution due to cluster formation (a,d), which after shaking are re-dissolved, thus recovering the initial spectra (b,e). As obvious from the slower and more uniform decrease in absorbance in the experiments with stirring (top), the mechanical agitation slows down/hinders cluster formation to some extent, however not sufficiently to cancel its impact preventing seeded supramolecular polymerization.

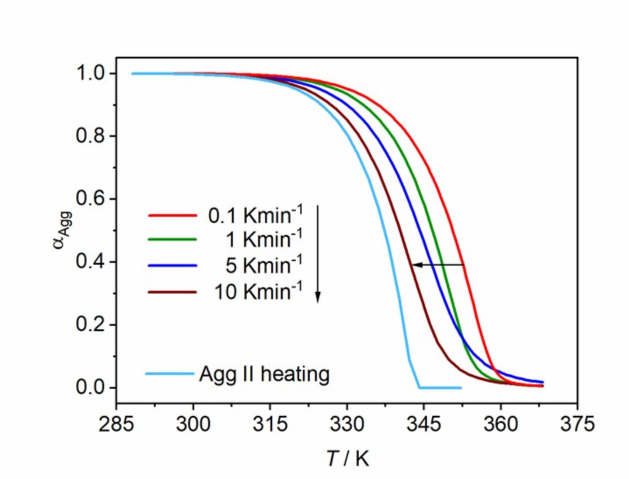

Figure S22: Cooling curves of **Agg I** (c = 5 x 10^{-6} M) for different cooling rates showing a slight increase of the thermal hysteresis. However, even at the fastest rate (10 $Kmin^{-1}$, highest kinetic influence), the onset of **Agg I** elongation never falls below the elongation temperature necessary to initiate the kinetic **Agg II** polymerization.

5. Calculation of the molar fraction of Agg I and Monomer *vs.* Temperature

The heating experiments of **Agg II** and the thermally induced transitions from **Agg II** to **Agg I** exhibited an isosbestic point and no intermediate appearance of the monomer spectra could be detected. This suggests a direct incorporation of every monomer which is released in the disassembly of **Agg II**, into **Agg I**. Consequently, the molar fraction of the monomer can be estimated by subtracting the degree of aggregation $\alpha'_{\mathrm{Agg\ I}}$ obtained for **Agg I** in heating experiments of **Agg I**:

$$\alpha_{Mono} = 1 - \alpha'_{Agg\ I} \qquad (1)$$

Furthermore, the sum of the molar fractions of all species (α_{total}) in multi-species processes is constant (= 1), which can be reorganized to give $\alpha_{\mathrm{Agg\ I}}$:

$$\alpha_{total} = 1 = \alpha_{Mono} + \alpha_{Agg\ I} + \alpha_{Agg\ II} \qquad (2)$$

$$\rightarrow \alpha_{Agg\ I} = 1 - \alpha_{Mono} - \alpha_{Agg\ II} \qquad (3)$$

As the degree of aggregation $\alpha_{\mathrm{Agg\ II}}$ is known from the heating experiments of **Agg II** ($\alpha_{\mathrm{Agg\ II}} = \alpha'_{\mathrm{Agg\ II}}$), the molar fraction of **Agg I** in the same experiment can thus be estimated *via* equation (4) which results from substitution of eq. (1) and $\alpha_{\mathrm{Agg\ II}} = \alpha'_{\mathrm{Agg\ II}}$ into eq. (3):

$$\rightarrow \alpha_{Agg\ I} = 1 - \alpha_{Mono} - \alpha_{Agg} \qquad (4)$$

The data used for this estimation of the molar fraction of the different species was the data obtained by the fits of the corresponding heating curves.

6. Experimental Part

6.1. Materials and Methods

General Procedures

All solvents were dried according to standard procedures. Reagents were used as purchased. All air-sensitive reactions were carried out under argon or nitrogen atmosphere.
For all spectroscopic measurements, spectroscopic grade solvents were used.

Colum Chromatography

Preparative column chromatography was conducted in self-packed glass columns of different sizes with silica gel (*Merck Silica 60*, particle size 0.04–0.063 nm).

NMR measurements

^{1}H and ^{13}C NMR spectra were recorded on a *Bruker Avance 400* (^{1}H: 400 MHz; ^{13}C: 100.6 MHz) and a *Bruker AV300* (^{1}H: 300 MHz; ^{13}C: 100.6 MHz). Additional 1D ^{1}H as well as 2D H,H COSY and 2D H,H ROESY spectra were recorded on an *Agilent DD2 500* (^{1}H: 500 MHz) and an *Agilent DD2 600* (^{1}H: 600 MHz) at a standard temperature of 298 K in deuterated solvents. The recorded spectra were referenced to the remaining resonance signals of the deuterated solvents The coupling constant J of the measured spin multiplets is given in Hertz (Hz) and the chemical shifts δ are given in reference to the chemical shift of trimethylsilane (0 ppm). Multiplicities for proton signals are abbreviated as *s*, *d*, *t*, *q* and *m* for singlet, doublet, triplet, quadruplet and multiplet, respectively.

Mass spectrometry (MS)

MALDI mass spectra were recorded on a *Bruker Daltronics Ultraflex ToF/ToF* or a *Bruker Daltronics Autoflex Speed* with a *SmartBeamTM* NdYAF-Laser with a wavelength of 335 nm. ESI mass spectra were measured on *a Bruker MicrOToF* system. The signals are described by their mass/charge ratio (*m/z*) in u.

UV-Vis spectroscopy

UV-Vis absorption spectra were recorded on a *JASCO V-770* or a *JASCO V-750* with a spectral bandwidth of 1.0 nm and a scan rate of 400 nm min^{-1} or on an *Agilent Cary 4000* with a spectral bandwidth of 2 nm at a scan rate of 600 nm min^{-1}. Glass cuvettes with an optical length of 1 cm, 1 mm and 0.1 mm were used. All measurements were conducted in commercially available solvents in spectroscopic grade.

Fluorescence spectroscopy

Fluorescence and excitation spectra were recorded on a *JASCO Spectrofluorometer FP-8500* in quartz cuvettes (*SUPRASIL®, Hellma*) with an optical length of 1 cm.

FT-IR spectroscopy

Solution and solid-state measurements were carried out using a *JASCO-FT-IR-6800* equipped with a CaF_2 cell with a path length of 0.1 mm.

Atomic force microscopy (AFM)

The AFM images were recorded on a *Multimode®8 SPM System* manufactured by *Bruker AXS*. The used cantilevers were *AC200TS* by *Oxford Instruments* with an average spring constant of 9 N m^{-1}, an average frequency of 150 kHz, an average length of 200 µm, an average width of 40 µm and an average tip radius of 7 nm. All samples were spin-coated from freshly prepared solutions onto an HOPG surface at a spin velocity between 2000 and 4000 rpm.

Dynamic Light Scattering (DLS)

DLS spectra were been recorded on a *CGS-3 Compact Goniometer System* manufactured by *ALV GmbH*, equipped with a HeNe laser with a wavelength of 632.8 nm (22 mW) and an *ALV/LSE-5004 Digital Correlator* by *ALV GmbH*.

Gel permeation chromatography (GPC)

Gel permeation chromatography was performed on a *Shimadzu prominence GPC system* equipped with two *Tosoh TSKgel* columns (*G2500H XL*; 7.8 mm I.D. x 30 cm, 5 µm; Part. No. 0016135) with CH_2Cl_2 as eluent. The solvent flow was set to be 1 mL/min. Detection was carried out *via* a *Shimadzu prominence SPD-M20A* diode array detector (DAD).

Elemental Analysis

Elemental analyses were carried out on a *Vario EL III Element Analyzer* by *elementar Analysensysteme GmbH*.

6.2. Synthesis and Characterization

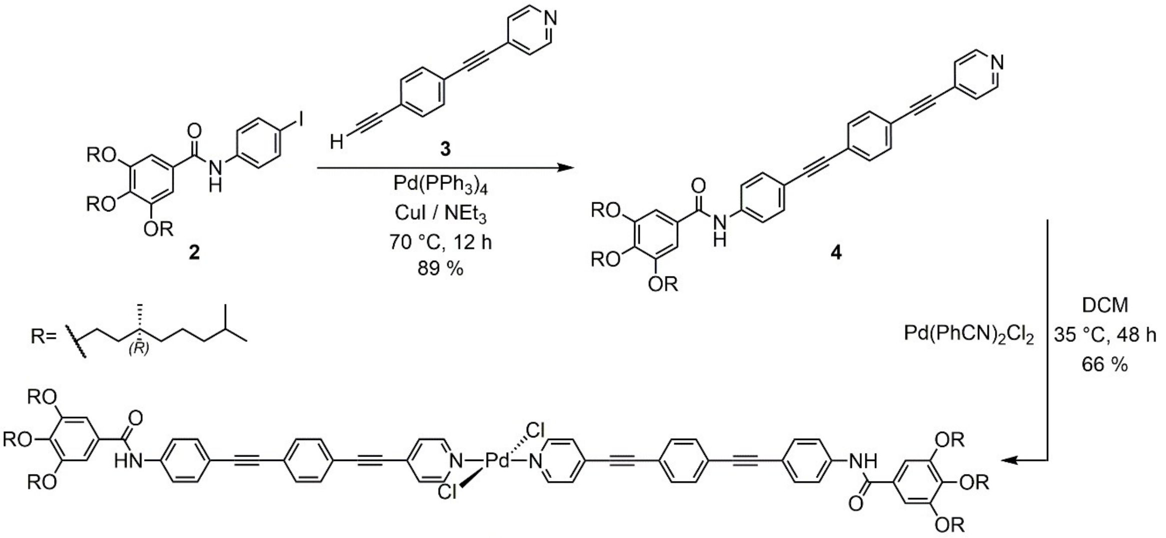

***(R)*-1**

Scheme S1: Synthesis route to obtain final compound *(R)*-**1**.

3,4,5-Tris((*(R)*-3,7-dimethyloctyl)oxy)-N-(4-iodophenyl)benzamide (**2**)[5] and 4-((4-ethynylphenyl)-ethynyl)pyridine (**3**)[6] were prepared following reported synthetic procedures and showed identical spectroscopic properties to those reported therein.

3,4,5-tris((*(R)*-3,7-dimethyloctyl)oxy)-N-(4-((4-(pyridine-4-ylethynyl)phenyl)ethynyl) phenyl)benzamide (4)

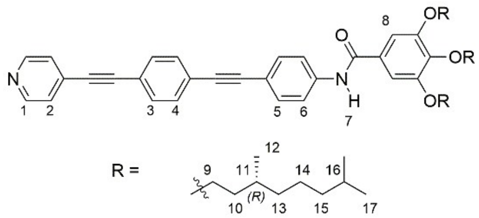

$C_{58}H_{78}N_2O_4$
867.27 g/mol

3,4,5-Tris((*(R)*-3,7-dimethyloctyl)oxy)-N-(4-iodophenyl)benzamide (**2**) (0.95 g, 1.20 mmol, 1 eq) tetrakis(triphenylphosphine)palladium(0) (81.71 mg, 0.071 mmol, 0.05 eq) and copper(I) iodide (13.47 mg, 0.071 mmol, 0.05 eq) were dissolved in a 1:1 mixture of degassed THF:NEt_3 (5 mL). The solution was stirred at 50 °C for 30 minutes. A solution of 4-((4-ethynylphenyl)-ethynyl)pyridine (**3**) (244 mg, 1.20 mmol, 1 eq) in a 1:1 mixture of degassed THF:NEt_3 (5 mL) was added dropwise. The solution was stirred at 70 °C for 12 hours. The solvents were removed *in vacuo* and the residue was dissolved in DCM and filtered over a thin layer (2 cm) of *Celite 545®*. The solvent was removed *in vacuo* and the crude product was purified by column chromatography (DCM:EtOAc 6:1) and several precipitation steps (DCM/MeOH + 1 % NEt_3) yielding the product as a colorless solid (0.93 g, 1.07 mmol, 89 %).

---

[5] F. Wang, M.A.J. Gillissen, P.J.M. Stals, A.R.A. Palmans and E.W. Meijer, *Chem. Eur. J.*, 2012, **18**, 11761-11770.
[6] A. Langenstroer, K. K. Kartha, Y. Dorca, J. Droste, V. Stepanenko, A. Rodrigo Q., M. R. Hansen, L. Sánchez and G. Fernández, *J. Am. Chem. Soc.,* 2019, **141**, 5192-5200.

^{1}H NMR (CDCl$_3$, 500 MHz): δ = 8.62 (*AA'XX'*, 2H, H_1), 7.78 (*s*, 1H, H_7), 7.68-7.64 (*m*, 2H, H_6), 7.57-7.53 (*m*, 2H, H_5), 7.53 (*s*, 4H, H_{3+4}), 7.39 (*AA'XX'*, 2H, H_2), 7.06 (*s*, 2H, H_8), 4.06 (*m*, 6H, H_9), 1.93-1,79 (*m*, 4H, H_{10}), 1.77-1.67 (*m*, 3H, H_{11}), 1.66-1.58 (*m*, 2H, H_{10}), 1.57-1.49 (*m*, 3H, H_{16}), 1.40-1.10 (*m*, 18H, H_{13-15}), 0.95 (*d*, 6H, $^3J_{HH}$ = 6.6 Hz, H_{12}), 0.93 (*d*, 3H, $^3J_{HH}$ = 6.6 Hz, H_{12}), 0.87 (*d*, 18 H, $^3J_{HH}$ = 6.7 Hz, H_{17}).

^{13}C NMR (CDCl$_3$, 126 MHz) δ (ppm) = 165.73, 153.50, 149.98, 141.95, 138.50, 132.73, 131.99, 131.72, 131.39, 129.77, 125.66, 124.42, 121.85, 119.91, 118.77, 106.01, 93.74, 91.80, 88.87, 88.47, 71.99, 68.01, 39.52, 39.42, 37.65, 37.50, 36.53, 30.01, 29.82, 28.14, 24.87, 22.86, 22.77, 19.75, 19.72.

HR-MS (MALDI-TOF, dithranol): calculated for $C_{58}H_{79}N_2O_4^+$ [M+H]$^+$: 867.6034,
found: 867.6025

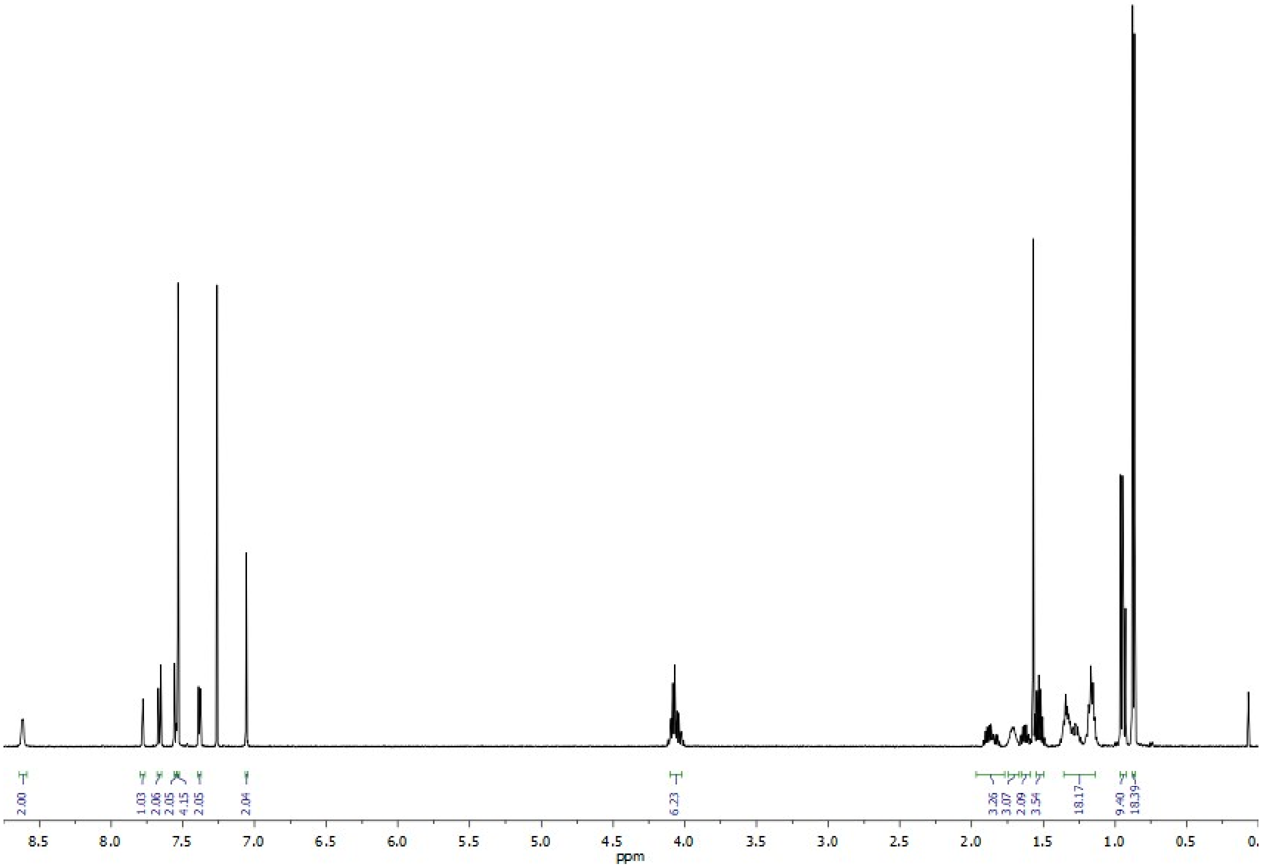

Figure S23: ^{1}H NMR (CDCl$_3$, 500 MHz) spectrum of compound **4**.

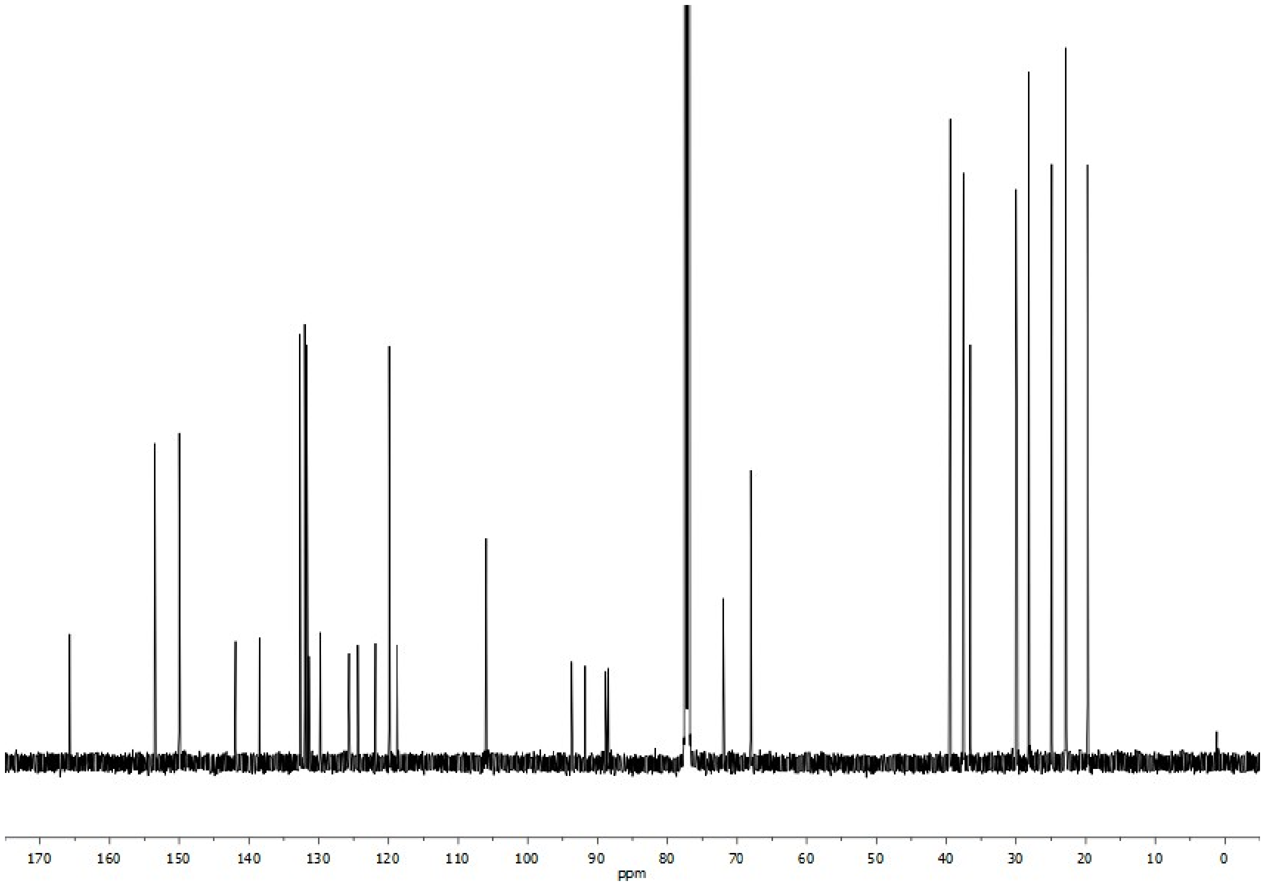

Figure S24: ^{13}C NMR ($CDCl_3$, 126 MHz) spectrum of compound **4**.

Palladium(II) Complex [PdL$_2$]Cl$_2$ (*(R)*-1)

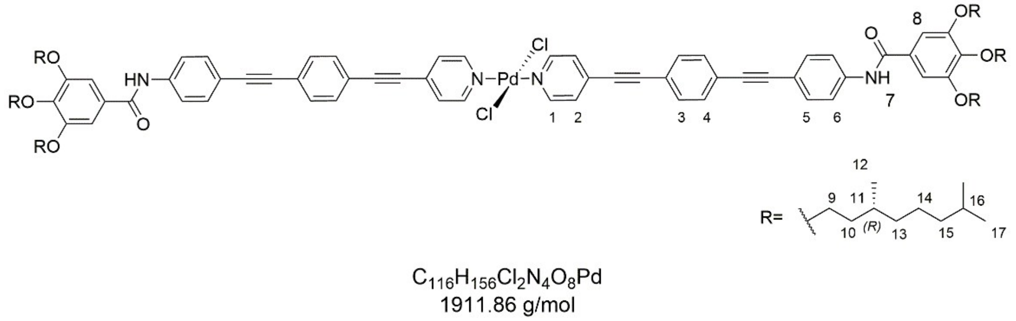

3,4,5-tris((*(R)*-3,7-dimethyloctyl)oxy)-N-(4-((4-(pyridin-4-ylethynyl)phenyl)ethynyl)phenyl)- benzamide (**4**) (50 mg, 57.56 μmol, 2.00 eq) was dissolved in 5 mL anhydrous, degassed DCM. A solution of bis(benzonitrile)palladium(II) dichloride (11.06 mg, 28.83 μmol, 1.00 eq) in 1 mL anhydrous, degassed DCM was added dropwise. The solution was heated to 35 °C and stirred for 48 hours. The solution was filtered through *Celite 545*® and rinsed with DCM. The solvent was removed *in vacuo* and the residue was dissolved in as few DCM as possible and precipitated with methanol. The process was repeated various times. The resulting yellow solid was subjected to gel permeation chromatography (GPC) to yield the pure product as a pale yellow solid (36.50 mg, 19.09 μmol, 66 %).

^{1}H NMR ($CDCl_3$, 500 MHz) δ (ppm) = 8.52 (*AA'XX'*, 4H, H_1), 8.1 (*s*, 2H, H_7), 7.66-7.64 (*m*, 4H, H_5), 7.49-7.46 z(*m*, 8H, H_{3+4}), 7.44-7.42 (*m*, 4H, H_6), 7.25 (*AA'XX'*, 4H, H_2), 7.12 (*s*, 4H, H_8), 4.11-4.01 (*m*, 12H, H_9), 1.91-1,79 (*m*, 8H, H_{10}), 1.75-1.65 (*m*, 6H, H_{11}), 1.64-1.56 (*m*, 4H, H_{10}), 1.55-1.48 (*m*, 6H, H_{16}), 1.40-1.10 (*m*, 36H, H_{13-15}) 0.97-0.91 (*m*, 18H, H_{12}), 0.87 (*d*, 36 H, $^3J_{HH}$ = 6.6 Hz, H_{17}).

^{13}C NMR (126 MHz, $CDCl_3$) δ (ppm) = 165.65, 153.20, 152.46, 141.52, 138.63, 134.28, 132.44, 132.06, 131.46, 129.52, 126.59, 125.05, 120.60, 119.65, 118.27, 105.92, 97.85, 92.26, 88.53, 86.88, 71.77, 67.69, 39.35, 39.26, 37.51, 37.49, 37.36, 37.33, 36.35, 29.86, 29.71, 27.97, 24.73, 24.72, 24.70, 22.69, 22.61, 22.59, 19.61, 19.58, 19.57.

Elemental Analysis: calculated for $C_{116}H_{156}N_4O_8Pd$: C: 72.88, H: 8.22, N: 2.93
found: C: 72.51, H: 8.53, N: 2.79

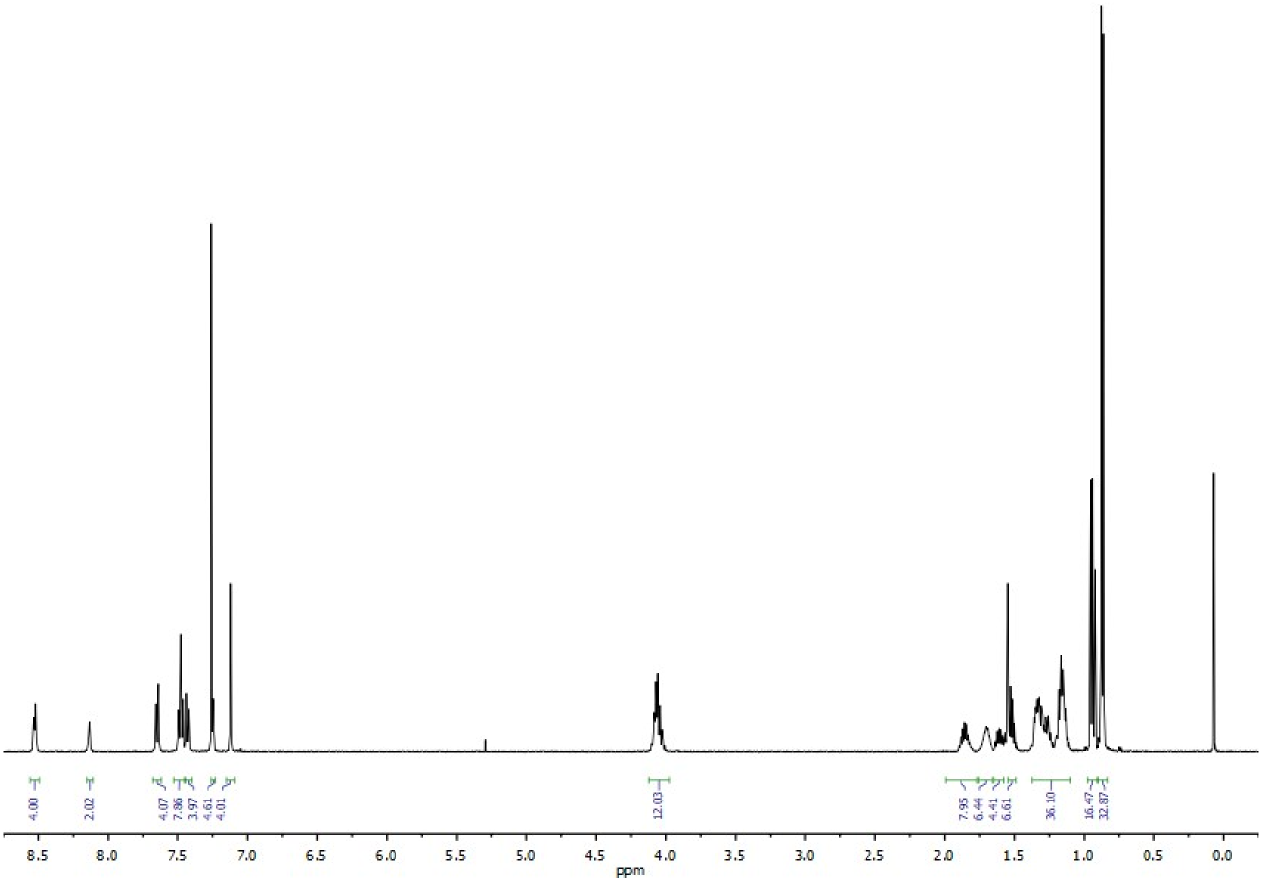

Figure S25: ^{1}H NMR ($CDCl_3$, 500 MHz) spectrum of compound *(R)*-**1**.

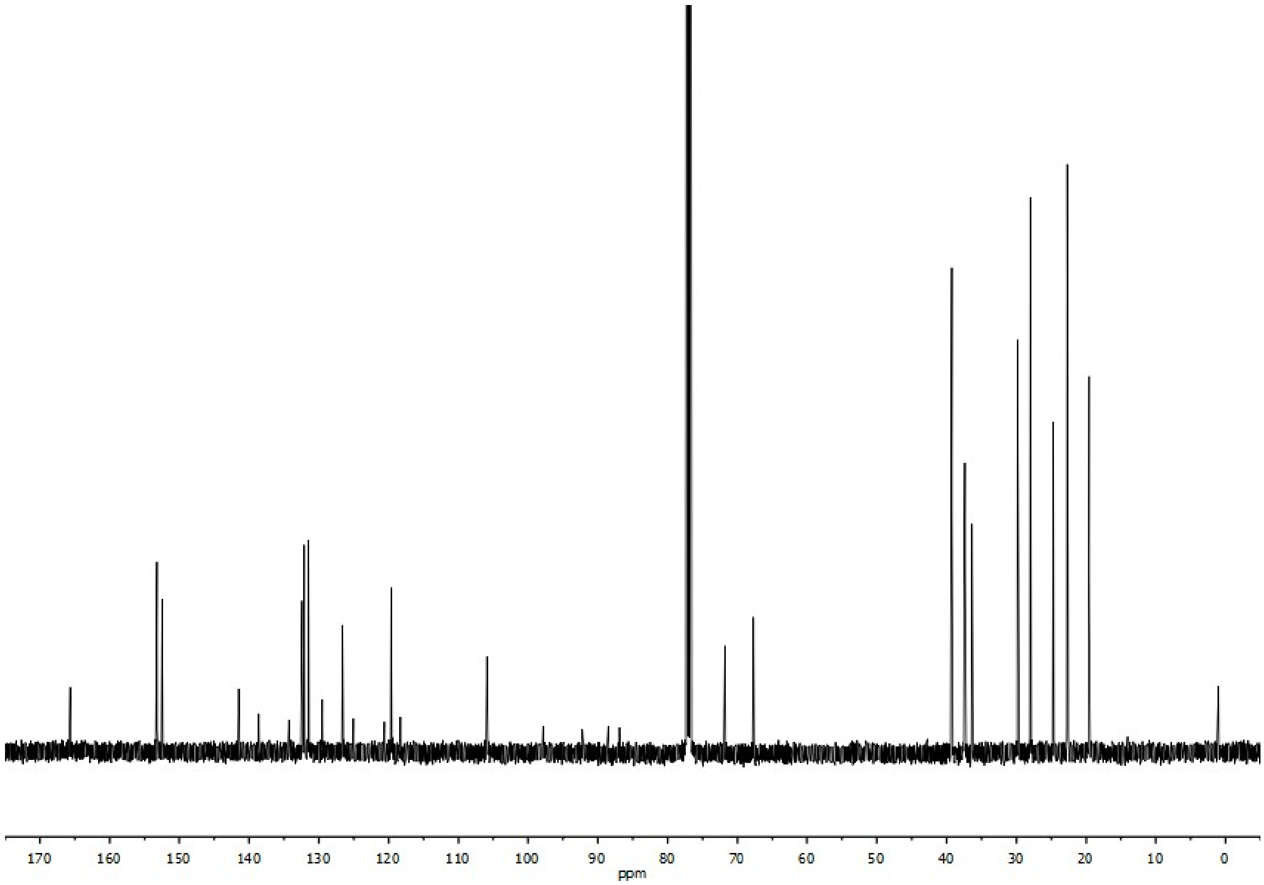

Figure S26: ^{13}C NMR ($CDCl_3$, 500 MHz) spectrum of compound *(R)*-**1**.
